# From Self-Esteem to Selflessness: An Evidence (Gap) Map of Self-Related Processes as Mechanisms of Mindfulness-Based Interventions

**DOI:** 10.3389/fpsyg.2021.730972

**Published:** 2021-11-22

**Authors:** Willoughby B. Britton, Gaëlle Desbordes, Rebecca Acabchuk, Sarah Peters, Jared R. Lindahl, Nicholas K. Canby, David R. Vago, Travis Dumais, Jonah Lipsky, Hannah Kimmel, Lauren Sager, Hadley Rahrig, Aya Cheaito, Pamela Acero, Jodi Scharf, Sara W. Lazar, Zev Schuman-Olivier, Rebecca Ferrer, Ethan Moitra

**Affiliations:** ^1^School of Public Health, Brown University, Providence, RI, United States; ^2^Department of Psychiatry and Human Behavior, Warren Alpert Medical School, Brown University, Providence, RI, United States; ^3^Athinoula A. Martinos Center for Biomedical Imaging, Massachusetts General Hospital, Harvard Medical School, Boston, MA, United States; ^4^Department of Psychological Sciences, University of Connecticut, Storrs, CT, United States; ^5^Department of Religious Studies and Clinical and Affective Neuroscience Lab, Brown University, Providence, RI, United States; ^6^Department of Psychology, Clark University, Worcester, MA, United States; ^7^Osher Center for Integrative Medicine, Vanderbilt University, Nashville, TN, United States; ^8^Department of Psychiatry, Massachusetts General Hospital, Harvard Medical School, Boston, MA, United States; ^9^Department of Psychiatry, Cambridge Health Alliance, Harvard Medical School, Cambridge, MA, United States; ^10^Basic Biobehavioral and Psychological Sciences Branch, National Cancer Institute, Bethesda, MD, United States

**Keywords:** mindfulness, meditation, self-related processes, mechanism, measurement

## Abstract

Self-related processes (SRPs) have been theorized as key mechanisms of mindfulness-based interventions (MBIs), but the evidence supporting these theories is currently unclear. This evidence map introduces a comprehensive framework for different types of SRPs, and how they are theorized to function as mechanisms of MBIs (target identification). The evidence map then assesses SRP target engagement by mindfulness training and the relationship between target engagement and outcomes (target validation). Discussion of the measurement of SRPs is also included. The most common SRPs measured and engaged by standard MBIs represented valenced evaluations of self-concept, including rumination, self-compassion, self-efficacy, and self-esteem. Rumination showed the strongest evidence as a mechanism for depression, with other physical and mental health outcomes also supported. Self-compassion showed consistent target engagement but was inconsistently related to improved outcomes. Decentering and interoception are emerging potential mechanisms, but their construct validity and different subcomponents are still in development. While some embodied self-specifying processes are being measured in cross-sectional and meditation induction studies, very few have been assessed in MBIs. The SRPs with the strongest mechanistic support represent positive and negative evaluations of self-concept. In sum, few SRPs have been measured in MBIs, and additional research using well-validated measures is needed to clarify their role as mechanisms.

## Introduction

Mindfulness-based interventions (MBIs), the most common of which are Mindfulness-based Cognitive Therapy (MBCT; Teasdale et al., 2002; Segal et al., 2013) and Mindfulness-based Stress Reduction (MBSR; Kabat-Zinn, 1982, 2013), are widely used to improve mental and physical health and to promote health behavior changes (Goldberg et al., 2021). Yet, as with other types of interventions, the mechanisms of MBIs are not well delineated. Consequently, MBIs may not be maximally optimized through targeting mechanisms of clinical change (Gu et al., 2015; Van Dam et al., 2018). Theoretical models suggest that self-related processes (SRPs) may be important mechanistic targets of MBIs (Goldin et al., 2009; Hölzel et al., 2011; Vago and Silbersweig, 2012; Carlson, 2013; Desbordes, 2019), but it is unclear to what extent these theories are supported by empirical evidence.

The primary goals of this evidence map are: (1) to identify which SRPs have been theorized to play a mediational role in MBIs; (2) to evaluate the extent to which these theories have been empirically investigated; and (3) to provide recommendations for future research. To do this, we first provide a conceptual framework in order to identify and differentiate the various types of SRPs that may be targeted through MBIs. We identify various SRPs and how they are theorized to operate as mechanisms in MBIs. For each theorized SRP, we evaluate the current state of evidence for MBI target engagement and assess to what extent SRP target engagement impacts outcomes (target validation). The review of the evidence provides commentary on the measurement of SRPs, highlights limitations in extant research, and identifies areas that require more clarification.

### Self-Related Processes

**FIGURE 1:**
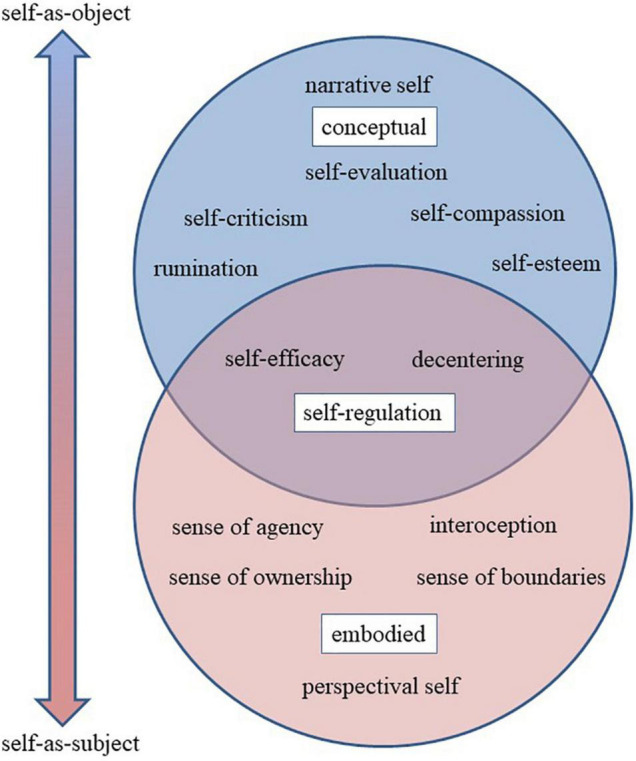
Schematic diagram of self-related processes.

Studying self-related processes is necessarily complex on account of involving the notion of self, which has long been recognized as a “mushy, muddle-headed construct without empirical referents” that encompasses multiple meanings (Westen, 1992) (p. 4). Indeed, various disciplines employ multiple terms in reference to similar conceptions of self and also employ the same (or similar) terms to refer to different SRPs –a conceptual morass known as the jingle-jangle fallacy (Roeser et al., 2006). Here we attempt to define and delineate various SRPs before querying how they are impacted by MBIs. SRPs have been defined as “processing requiring one to evaluate or judge some feature in relation to one’s perceptual image or mental concept of oneself,” which the authors differentiate from embodied, pre-reflective self-specifying processes “that specifies the self as subject and agent by implementing a functional self/non-self distinction”(Christoff et al., 2011b, p.104). However, the relationship between some pre-reflective self-specifying processes and evaluative self-related processes may less distinct and more dynamic (Balconi, 2010; Gallagher and Daly, 2018). For the sake of simplicity, in this review we use SRPs in a more general sense to identity a range of potential mechanisms of MBIs, whether conceptual and evaluative processes, pre-reflective embodied processes, or processes of self-regulation. While recognizing that there remain many debates about to delineate and differentiate them (Christoff et al., 2011a; Northoff, 2011), we organize the SRPs investigated in this evidence map along a continuum from more embodied “self-as-subject” processes to more conceptual “self-as-object” processes (Figure 1).

Perhaps the most fundamental aspect of the self-as-subject is the perspectival self, or the sense that our experience is situated from a specific, first-person perspective or vantage point (Zahavi, 2005, 2012). Other terms that have been used for the more basic embodied senses of self include core self, proto-self (Damasio, 2010) and minimal self (Gallagher, 2000). The broader concept of minimal self often includes additional pre-reflective processes central to embodiment. Interoception, or body awareness, is supported by the sense of ownership, or the extent to which thoughts, emotions, and sensations present themselves as “me” or “mine” vs. “not me” or “not mine.” The sense of agency – or self as agent, doer, or controller – can be described as the sense of ownership extended to actions (Gallagher, 2000), although agency and ownership can also be empirically differentiated (Tsakiris et al., 2007).

In contrast to the pre-reflective self-as-subject, the upper circle in Figure 1 represents senses of self supported by conceptual, narrative, and evaluative processes. The narrative self is often described as the autobiographical story of me as a person with a temporally extended identity, including the conception of past and future selves, multiple imagined selves (Gallagher, 2000). The narrative self also includes various social identities, group memberships and associated roles. Many key psychological constructs such as self-esteem and self-evaluation could be considered more self-as-object SRPs. Negative self-evaluations include self-criticism and rumination, whereas positive evaluations include self-esteem, self-worth, and self-praise. Many forms of psychotherapy focus on decreasing rumination, increasing self-esteem, and shifting the valence of the self-concept from negative to positive (Kyrios et al., 2016).

Lastly, we identify SRPs that function as self-regulation skills, specifically decentering and self-efficacy. Self-efficacy, for example, is the perceived capability of an imagined future self to perform a target behavior (Bandura, 1977) and is a type of self-evaluation that relates not only to imagined qualities, capacities, and goals of the conceptual self or identity, but also to the embodied and agentic self and its capacity to engage in action – especially goal-related behavior. As such, we represented these SRPs at the intersection of bodily senses of self and conceptual senses of self (where the two circles overlap in Figure 1).

### Self-Related Processes and Mindfulness

Mindfulness-based interventions operate at the confluence of multiple disciplines, including clinical, health, cognitive, and positive psychology, as well as Buddhist theories of mind. As products of clinical and health psychology, MBIs might be expected to promote any number of SRPs associated with self-concepts and self-regulation. However, in line with the Buddhist theories from which they are derived, many contemporary models for the mechanisms of mindfulness also propose that MBIs affect (e.g., attenuate or transform) many, if not most, dimensions of selfhood, not just conceptual ones (Hölzel et al., 2011; Teasdale and Chaskalson, 2011a, b; Vago and Silbersweig, 2012; Kabat-Zinn, 2013; Brown et al., 2015; Lutz et al., 2015). Indeed, many theories hypothesize that changes in more embodied self-specifying processes, including ownership, agency, and interoception underlie the beneficial effects of mindfulness meditation (Olendzki, 2006; Kabat-Zinn, 2013; Brown et al., 2015; Hadash et al., 2016). However, exactly which aspects of the self are being transformed, attenuated, or transcended through meditation have been subject to considerable debate in historical sources across Buddhist traditions (Albahari, 2002), as well as in contemporary humanistic and experimental contexts (Hölzel et al., 2011; Vago and Silbersweig, 2012; Ostafin et al., 2015; Yaden et al., 2017; Desbordes, 2019). The following evidence map introduces a comprehensive framework the different types of SRPs that have been investigated in MBIs, describes how they are theorized to function as mechanisms, and evaluates the strength of empirical evidence for SRPs target engagement and target validation.

## Review of Self-Related Processes Constructs as Mechanisms of Mindfulness-Based Interventions

### Review Methods

Self-related processes are theorized as key mechanisms of MBIs. According to the experimental medicine approach, hypothesized intervention targets must meet several other basic criteria to qualify as a mechanism (Kazdin, 2007; Onken et al., 2014; Riddle and Science of Behavior Change Working Group, 2015). In addition to theory (target identification), validated measures or assays that accurately represent the theorized SRP construct must exist or be created. Once created and validated, the SRP assay must be reliably engaged by mindfulness meditation training (target engagement), and the degree of SRP target engagement must account for the change in desired outcomes (target validation).

The current paper is an evidence map that uses a multidimensional methodology to synthesize the evidence. An evidence map is generally a type of knowledge synthesis that maps key concepts, types of evidence, and gaps in knowledge. While unrestricted to a specific methodology, depending on goals and resources an evidence map typically includes: a review of key documents; consultation with stakeholders or experts; specific inclusion criteria and a systematic method of search; data extraction; and critical evaluation (Miake-Lye et al., 2016; Snilstveit et al., 2016). Compared to other types of reviews, evidence maps, or more pointedly “evidence gap maps” are particularly well-suited to “identify broad trends in a particular area of research and highlight key shortcomings in the existing evidence base” (Snilstveit et al., 2016, p. 123). One defining feature of an evidence (gap) map is the visual representation evidence in an accessible, user-friendly figure or graph in order to most effectively highlight “absolute gaps where there are few or no studies…where new primary studies or systematic reviews can add most value” (Miake-Lye et al., 2016; Snilstveit et al., 2016, p. 123).

A detailed description of our methodology can be found in the Supplementary Materials (see Supplementary Methods) but is described briefly here. We initiated and anchored the evidence map with a systematic review of target engagement in RCTs of standard MBIs since RCTs present the strongest evidence for causal inference of the effects of mindfulness meditation training on specific outcomes. The target identification phase was performed by an expert panel of five mindfulness researchers with broad knowledge of SRPs. In addition to search terms beginning with or including the word “self,” the panel generated 30 SRP constructs to serve as search terms, namely: *agency, body awareness, decentering, disidentification, ego, embodiment, experiential self, identity/identification, interoception, meta-awareness, narrative self, observing self, perceived control, reperceiving, self-awareness, self-compassion, self-criticism, self-distancing, self-efficacy, self-esteem, self-loathing, self-monitoring, self-praise, self-referential processing, self-regulation, self-related rumination, self-worth, sense of agency, sense of control*, and *sense of ownership*.

Eligible studies were English-language RCTs of standard Mindfulness-Based Stress Reduction (MBSR) and Mindfulness-Based Cognitive Therapy (MBCT) with one or more active and/or inactive control condition(s), with adult participants (age 18 or older) from both clinical and non-clinical samples. Of the 10,802 studies identified, only 14 standard MBI RCTs assessed SRPs. In addition, only five of the 30 SRP constructs generated by the expert panel had been measured within an RCT of a standard MBI, namely: self-evaluation, self-compassion, rumination, self-esteem, and self-efficacy.

Because the initial review demonstrated that very few SRPs had been measured in the context of standard MBI RCTs, we adapted our mapping approach to match the inchoate state of the research. The expert panel agreed to specifically include (and allow less rigorous inclusion criteria for) SRP constructs that have been theorized as central or key mechanism of MBIs, specifically *interoception, decentering, selflessness and self-transcendence* (Hölzel et al., 2011; Vago and Silbersweig, 2012). This allowed us to provide both an evidence map and an evidence gap map for a wider range of SRPs than yielded by the initial review.

Thus, using the experimental medicine framework (Kazdin, 2007; Onken et al., 2014; Riddle and Science of Behavior Change Working Group, 2015) we created an “evidence gap map” (Snilstveit et al., 2016) by describing other mindfulness meditation studies that investigate SRPs and organized them hierarchically according to strength of evidence. The highest level of evidence are meta-analyses of multiple MBI RCTs where an SRP fully mediates desired outcomes (Kazdin, 2007), although few SRPs met this criteria (Gu et al., 2015; van der Velden et al., 2015; Alsubaie et al., 2017). If no meta-analyses of MBI RCTs were available, then single RCTs of standard, then modified MBIs were described. If the target had not been tested in a longitudinal RCT, then brief experimental meditation induction studies were permitted. If induction studies were absent, then cross-sectional studies of meditators vs. non-meditators were reviewed.

**FIGURE 2:**
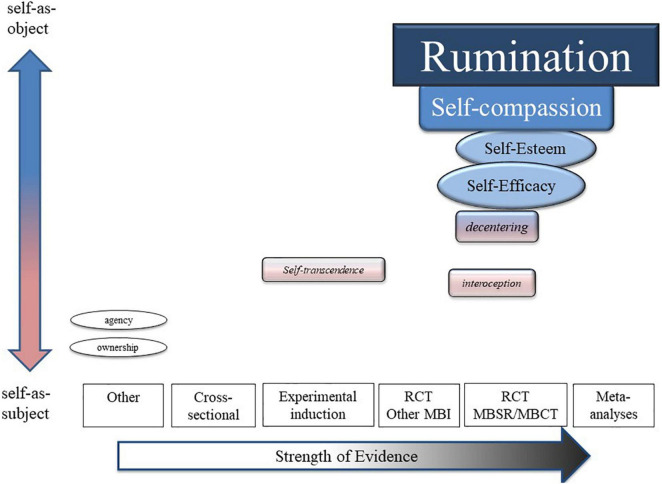
Evidence map.

The review of each construct included four parts: (1) a definition and how it is theorized to function as a mechanism of MBIs (target identification); (2) description of target engagement by mindfulness meditation training according to a hierarchical levels of evidence (meta-analysis of MBI RCTs > MBI RCTs > modified MBI RCTs > induction studies > cross-sectional studies); (3) description of target validation, or the degree to which target engagement account for desired outcomes, using similar levels of evidence above, with Kazdin’s full mediation criteria at the top (Kazdin, 2007); and (4) commentary to discuss caveats and other limitations. Figure 2 provides a user-friendly visual presentation of the evidence and evidence gaps, which are further detailed in the following sections. A table of all SRP studies and constructs can be found in the Supplementary Material (see Supplementary Table 1).

## Results: an Evidence (Gap) Map of Self-Related Processes as Mechanisms for Mindfulness-Based Interventions

### Conceptual Self-Related Processes

Conceptual SRPs constituted the highest level of evidence as mechanisms of MBIs. Five SRP constructs were organized under two types of self-evaluation: negative and positive. Specifically, rumination, dysfunctional attitudes and negative automatic thoughts were categorized under negative self-evaluation, while self-compassion and self-esteem were categorized under positive self-evaluation.

#### Negative Self-Evaluation

##### Rumination

Rumination could be described as a form of depressive negative automatic thinking (Teasdale et al., 1995). However, it is better conceptualized as a form of repetitive negative thinking focused on the past (Nolen-Hoeksema et al., 2008). This is in contrast to worry, which has been described as repetitive negative thinking focused on the future (Papageorgiou and Wells, 1999). Mindfulness theory suggests that MBIs support healthy emotion regulation skills by undermining the ruminative process (Perestelo-Perez et al., 2017). Thus, individuals are able to mindfully accept or tolerate negative thoughts, thereby disengaging from rumination (Teasdale et al., 1995).

A meta-analysis by Gu et al. (2015) showed a significant combined association (*r* = 0.31, *p* < 0.001) between MBIs and rumination in six studies of MBCT and MBSR. In another meta-analysis focused mainly on MBCT for depressed patients, pooled results showed significant reductions in rumination following MBCT compared to treatment-as-usual (*g* = 0.59, 95%CI: 0.41, 0.77; Perestelo-Perez et al., 2017). A review of brief MBIs also showed significant effects of MBI reducing negative affectivity, including rumination (*g* = 0.21, 95%CI: 0.12, 0.29; Schumer et al., 2018). Overall, a moderate amount of research supports the engagement of rumination by MBIs.

In terms of target validation, accumulating research supports rumination as a pathway to improved outcomes. A meta-analysis of mechanisms of MBIs found “consistent and moderate evidence” for rumination as a potential mediator of MBCT and MBSR on mental health outcomes (Gu et al., 2015). A meta-analysis of mechanisms of MBCT trials (Perestelo-Perez et al., 2017) showed that in studies with medium to strong effects on rumination (*k* = 6), a significant reduction in depression scores was facilitated by changes in rumination (*g* = 0.55, 95%CI: 0.15, 0.95). In the subgroup of studies that found weak effects on rumination (*k* = 6), there was a reduced effect on depression (*g* = 0.29; 95%CI: 0.00, 0.58). In a narrative review focused on mediation studies, results showed that rumination was successfully engaged in MBCT and MBSR and that this engagement led to improved health states (Alsubaie et al., 2017). These results were compiled from five RCTs and other controlled trials for a variety of adults with physical health problems that examined rumination as a mediator. In sum, data show that rumination is a validated target of MBIs that consistently leads to improved mental health outcomes.

##### Dysfunctional Attitudes

Dysfunctional attitudes were first described in Beck’s cognitive theory of depression (Beck, 1967, 1976) and are thought to constitute important aspects of cognitive vulnerability to depression (Segal et al., 2006). Overall, dysfunctional attitudes are characterized by negative, rigid, and extreme assumptions and beliefs about self-worth and typically involve conditional standards in areas of evaluation, perfectionism, and interpersonal approval (Zuroff et al., 1999). It is theorized that MBIs can engage dysfunctional attitudes by teaching individuals to: (1) identify destructive contents and habitual patterns of the mind at an early stage; and (2) relate and process this information in a non-judgmental way that reduces self-critical evaluation (Teasdale et al., 2002; Segal et al., 2013).

A number of RCTs investigated the impact of MBIs on dysfunctional attitudes, with all studies showing positive findings. These include RCTs of standard MBCT in a variety of adult samples with physical health problems, including cancer (Mehdipour et al., 2017), hypertension (Hasanzade and Khalatbari, 2017), and fertility problems (Ebrahimi et al., 2019). One study (Kaviani et al., 2012) found greater reductions in dysfunctional attitudes in MBCT compared to an inactive control (*d* = 0.32 immediately following the 8-week intervention; *d* = 0.65 and 0.79 at 13 and 34 week follow-up, respectively). In another RCT among adults with Bipolar I disorder (Docteur et al., 2020), dysfunctional attitudes were also significantly reduced in MBCT compared to a waitlist control. To our knowledge, only one RCT examined MBSR’s effects on dysfunctional attitudes (Ramel et al., 2004). This study also showed significant effects relative to a waitlist control. These findings are strengthened by results from non-MBCT/MBSR brief MBIs that also show impacts on dysfunctional attitudes, including among students with subclinical depression (Kaviani et al., 2012) and in an experimental paradigm with university students (Kuehner et al., 2009). Taken together, these data show that standard MBIs can elicit positive changes in dysfunctional attitudes compared to waitlists.

Overall, scant MBI research has attempted to validate dysfunctional attitudes as a mechanism leading to improved outcomes (e.g., depressive symptoms). A systematic review (Alsubaie et al., 2017) examined purported mediational MBI mechanisms, including dysfunctional attitudes. However, this review only identified one controlled trial in which dysfunctional attitudes were assessed (Jermann et al., 2013). While participation in MBCT did lead to reductions in dysfunctional attitudes, compared to usual care in an outpatient treatment setting, no assessment of how this change might lead to changes in outcomes (e.g., depressive symptoms) was conducted. More research is needed to validate changes in dysfunctional attitudes as they relate to outcomes.

##### Negative Automatic Thoughts

Negative automatic thoughts also come from Beck’s cognitive theory (Beck, 1967, 1976). These entail systematic information-processing biases that emerge as streams of consciousness, impacting assumptions about oneself, others, and the future. Although initially linked to depressive states, negative automatic thoughts are associated with numerous psychiatric conditions, including anxiety (Clark and Steer, 1996) and eating disorders (Jones et al., 2007). As with dysfunctional attitudes, MBIs are theorized to impact negative automatic thoughts by facilitating metacognitive awareness, thus setting the stage for non-judgmental informational processing to reduce their frequency and intensity (Teasdale et al., 2002; Segal et al., 2013).

No systematic reviews or meta-analyses have examined MBIs’ impact on negative automatic thoughts. MBIs led to reduced negative automatic thoughts in RCTs with college students (Kaviani et al., 2012), patients with cancer (Mehdipour et al., 2017) and, clinically depressed adults (Hofheinz et al., 2020). Yet, more research is needed to validate how engagement of negative automatic thoughts might lead to beneficial outcomes, such as improved psychological states.

#### Positive Self-Evaluation

##### Self-Compassion

Self-compassion is one of the mostly widely theorized and tested SRP mechanisms of change in MBIs (Kuyken et al., 2010; Van Dam et al., 2014). Self-compassion involves “treating yourself with the same kindness, concern and support you would show to a good friend” (Neff and Dahm, 2015). Self-compassion overlaps with core components of mindfulness in that both involve non-judgmental awareness and acceptance. Self-compassion recognizes that imperfection is part of the shared human experience and emphasizes the importance of responding with kindness when facing challenges and difficulties in life. Self-compassion, as proposed by Neff (2003), entails three main components: (1) self-kindness – being kind and understanding toward oneself in instances of pain or failure rather than being harshly self-critical; (2) common humanity – perceiving one’s experiences as part of the larger human experience rather than seeing them as separating and isolating; and (3) mindfulness – holding painful thoughts and feelings in balanced awareness rather than over-identifying with them.

Overall, there is sufficient evidence to suggest MBIs successfully engage self-compassion. Two meta-analyses in clinical (Wilson et al., 2019) and non-clinical (Wasson et al., 2020) samples found increases in self-compassion after MBIs compared to inactive controls. A recent meta-analysis (Golden et al., 2021) of 22 MBIs found a medium pre-post change in self-compassion in MBIs compared to control conditions (*g* = 0.60), but noted that the effect was not consistent across studies. In addition, increases in self-compassion are not unique to or superior in MBIs. Evidence from active controls suggests self-compassion may also improve with other behavioral interventions such as exercise, yoga or biofeedback (Kirby and Gilbert, 2019).

Preliminary but insufficient evidence for self-compassion as a mechanism of change for mental health and wellbeing outcomes in MBIs was found in earlier reviews, including Gu et al.’s (2015) meta-analysis, and van der Velden et al.’s (2015) review on MBCT for recurrent major depressive disorder, and Khoury et al.’s (2013) meta-analysis of MBSR for healthy individuals. A more recent meta-analysis by Golden et al. (2021) assessed correlations between change in self-compassion and other mental health outcomes in non-clinical populations; the correlation between self-compassion and stress was not significant. Although there was insufficient power to evaluate the relationship between self-compassion with anxiety or depression, they noted that the relationship was not consistent, with “two studies showing an increase in self-compassion without a significant decrease in depression or anxiety, and two studies exhibiting the opposite pattern.” The authors concluded that there was “inconsistent support for self-compassion as a mediating factor in change” (p. 43).

##### Self-Esteem

Self-esteem refers to a person’s overall sense of self-worth or personal value, or a combination of “worthiness” and “competence” in areas of value to the individual (Mruk, 2013). This concept refers to how a person judges oneself as valuable, significant, and capable (Coopersmith, 1967). Self-esteem is generally considered to be an enduring trait, as opposed to a temporary state or mood. Low self-esteem is associated with greater negative self-related processing, including rumination (Fennell, 2004). Thus, mindfulness is theorized to improve self-esteem by reducing negative self-referential thought patterns and improving acceptance, positive affect and self-compassion (Randal et al., 2015). As a result, multiple health behavior change models and interventions include self-esteem as a key mechanistic target (Mann et al., 2004).

Self-esteem was assessed in only one study that we identified to be of the highest methodological quality, Jazaieri et al. (2012), which found that MBSR had a similar effect as the active control intervention (aerobic exercise) and significantly outperformed the inactive control. A previous systematic review (*k* = 17), found significantly greater increases in self-esteem in the MBI than in the control group in 6 of the 12 studies rated strong for their study design (Randal et al., 2015). A meta-analysis of compassion-based interventions found significant within-group improvements on self-esteem (*g* = 0.56; 95%CI: 0.19, 0.93; *k* = 8), but between group results were non-significant. Although potentially engaged by MBIs, self-esteem has yet to be investigated as a mechanism of MBIs on any outcome.

### Self-Regulation Skills

#### Self-Efficacy

Self-efficacy reflects confidence in one’s ability to exert control over one’s motivation, behavior, and social environment (Bandura, 1977, 1986, 1997). General self-efficacy is a measure of optimistic beliefs and confidence about one’s capacity to handle stressful or demanding situations or to exert control over symptoms and environment (Schwarzer and Jerusalem, 1995). Task-specific self-efficacy refers to confidence in one’s capacity to act in a specific situation and is a more relevant predictor of behavior than general self-efficacy (Bandura, 1982, 1997). In the context of MBIs, the emphasis on acceptance of present-moment experience through a non-interfering, non-fixing, allowing stance is expected to engender improved confidence in one’s self-efficacy (Abba et al., 2008; Hargus et al., 2010).

Accumulating reviews demonstrate that MBIs can engage self-efficacy. In a recent meta-analysis of controlled trials of 19 “mindfulness programs” for health profession students, two studies examined self-efficacy (McConville et al., 2017). MBIs significantly engaged self-efficacy compared to attention-matched controls (SMD = −0.82, 95%CI: −1.15, −0.49). In a small scoping review of MBIs for adults with rheumatoid arthritis (DiRenzo et al., 2018), one study was identified that showed significant engagement of self-efficacy. Lastly, in another narrative review, Emerson et al. (2017) showed that five MBIs targeting teacher self-efficacy did reliably elicit positive changes in teaching self-efficacy.

Further evidence of target engagement comes from a variety of RCTs in clinical and non-clinical samples. For instance, in two studies that used variations of MBSR, results showed improved maternal self-efficacy among breastfeeding women when compared to a waitlist (Perez-Blasco et al., 2013) and improved child-birthing self-efficacy among pregnant women (Zarenejad et al., 2020). In adults with chronic low back pain, results showed that MBSR significantly improved pain-related self-efficacy; however, the active comparison of CBT also led to improved self-efficacy (Turner et al., 2016). Overall, results show that MBIs can engage self-efficacy at a general or a more specific task- or condition-level. However, to our knowledge, no research has attempted to validate self-efficacy as a mechanism of change leading to improved outcomes in MBI. Further research is needed in this area.

#### Decentering and Related Constructs

A construct called “decentering” has emerged as a central hypothesized mechanism of MBIs (Bernstein et al., 2015, 2019; Vago and Zeidan, 2016; Dunne et al., 2019). Here we review the evidence for decentering as a mechanism in MBIs based on current theories (Hölzel et al., 2011; Bernstein et al., 2015; Lutz et al., 2015). In general, decentering refers to “the ability to observe one’s thoughts and feelings as transitory events in the mind that do not necessarily reflect reality, truth or self-worth and not necessarily important and do not require particular behaviors in response” (Sauer and Baer, 2010, p 35). However, the construct of decentering has different meanings depending on therapeutic tradition and other influences. The importance of observing and identifying thoughts as thoughts first emerged in cognitive therapy for depression where clients learn to test or even challenge negative thoughts (Safran, 1990; Safran and Segal, 1990).

In MBIs, decentering has taken on several different meanings. Drawing from the Buddhist teachings of not-self (*anatta*), MBIs expand the disidentification process to include not just negative thoughts but *all* thoughts, and *all* internal experiences, including emotions and body sensations. According to the *Handbook of Mindfulness*, disidentification is “fundamental to the effects of [mindfulness training] on positive functioning” (Brown et al., 2015, p. 318). Similarly, the creator of MBSR, Jon Kabat-Zinn, asserts that understanding the Buddha’s teaching that “nothing is to be clung to as I me or mine” is the key to MBSR’s benefits (Kabat-Zinn, 2010). “Disidentifying from the entire play of inner experience” (Kabat-Zinn, 2013, p. 297), including the body, is explicitly described as part of mindfulness practice in MBI curricula and in other mindfulness-related literature (Olendzki, 2006; Brown et al., 2015).

Researchers describe decentering as being comprised of several overlapping components or processes including, meta-awareness, dereification, disidentification, non-reactivity, and self-as-context (Fresco et al., 2007; Bernstein et al., 2015, 2019). Meta-awareness is awareness of the contents and processes of internal experience, thinking, feeling and perceiving. Dereification is the degree to which thoughts, feelings, and perceptions are phenomenally interpreted as mental processes rather than as accurate depictions of reality: it is seeing “thoughts as simply events in the mind with no *a priori* truth value” (Lutz et al., 2015, p. 647). Disidentification is the experience of internal states as separate from one’s self (e.g., “I experienced myself as separate from my changing thoughts and feelings”) (Bernstein et al., 2015).Non-reactivity may refer to “reduced effects of thought content on other mental processes” (e.g., attention, emotion, cognitive elaboration, motivation, motor planning) or to improved control over emotions and overt (re)actions. Self-as-context refers to an “observing self” or “I” that can serve as a “constant and stable […] place where content is observed” (Ciarrochi et al., 2010, p. 56). In addition to undermining the validity or truth of the thought and its relationship to action, clients are taught to disidentify with narrative and conceptual senses of self and to identify instead with “self-as-subject” processes referred to variously as “observing self,” “self-as-context,” “witness consciousness” and “awareness” (Bernstein et al., 2015).

Although no meta-analysis of decentering as a mechanism of MBIs has been conducted, several RCTs of MBSR or MBCT in clinical samples have measured target engagement. Gayner et al. (2012) measured decentering before and after MBSR or treatment-as-usual (TAU) in gay men living with HIV. While the treatments did not differ in clinical outcomes, increases in decentering were associated with improvements in anxiety, depression and PTSD, and scores increased significantly more in the MBSR group than through TAU. In a subsample from a larger parent study in GAD patients (Hoge et al., 2013), increases in decentering mediated decreases in anxiety but not worry in MBSR compared to stress management education (Hoge et al., 2015). Bieling et al. (2012) measured decentering in an RCT comparing MBCT plus antidepressant taper to antidepressant maintenance or placebo taper in remitted depressed individuals. While both measures increased in MBCT more than both control conditions, only increases in one measure were associated with improved depression at a 6-month follow-up. Finally, in a study comparing MBCT to cognitive therapy, decentering scores increased significantly for both groups of remitted depressed patients. Those who relapsed 24 months later had lower decentering scores (Farb et al., 2018). In a follow-up analysis in the same study, post-treatment use of the skills learned in treatment predicted increases in a composite decentering variable comprised of mindfulness, self-compassion and well-being scales, which in turn predicted relapse prophylaxis (Segal et al., 2019). Despite these generally supportive results related to MBCT and decentering, both analyses found that decentering could be produced by many types of skills and treatment and is not specific to mindfulness training.

In summary, there is some evidence that decentering mediates some of the therapeutic outcomes of MBIs. However, it is unclear what exactly is responsible for the benefits. Based on approaches used to measure decentering, the strongest evidence for decentering comes from disidentifying from negative self-evaluative thoughts, similar to cognitive therapy (Sauer and Baer, 2010). In contrast, there is currently no evidence that the benefits of MBIs come from a “more drastic disidentification” (Hölzel et al., 2011, p. 547) from all phenomena one takes to be a “me,” or from “the entire play of inner experience” (Kabat-Zinn, 2013, p. 297). Given that disidentification from thoughts, emotions and body sensation is also characteristic of dissociation, depersonalization, and other alterations in senses of self that are associated with impairment in functioning, it will be important for both MBI providers and researchers to be clear about the intended scope of decentering techniques (Phillips et al., 2001; Britton, 2019; Lindahl and Britton, 2019).

### Embodied Self-Related Processes

#### Interoception and Related Constructs

Interoception refers to the process of sensing, interpreting and integrating signals originating from inside the body (Sherrington, 1907; Craig, 2002; Mehling et al., 2009; Ceunen et al., 2016; Khalsa and Lapidus, 2016). However, there are debates concerning the scope of this concept. In its narrow sense, interoception is defined as the awareness of internal bodily signals, such as heart beat, breath, thirst, hunger, and pain, related to maintenance of homeostasis (Craig, 2003). Yet, the definition of interoception has evolved over the past two decades to become more comprehensive (Farb et al., 2015; Ceunen et al., 2016), incorporating an understanding of the reciprocal and iterative neurobiological processes of sensation, experience, and expectation (Barrett and Simmons, 2015); this broad definition of interoception goes beyond pure body sensation representations and includes how individuals interpret and react to these sensations (Ceunen et al., 2016; Khoury et al., 2018). In order to clarify conceptual vagueness regarding different aspects of this broader definition of interoception, Garfinkel et al. (2015) defined and distinguished between *interoceptive accuracy*, the correct monitoring of bodily sensations using objective measures, *interoceptive sensibility*, self-reported beliefs and subjective confidence about awareness of one’s internal bodily state, and *interoceptive awareness*, the correspondence between subjective and objective measures of internal bodily state. A further elaboration of this theory distinguishes between interoceptive accuracy and *interoceptive attention*, the degree to which interoceptive signals are the object of attention, both of which have been measured using self-report and objective measures (Murphy et al., 2020).

In MBIs, mindfulness training typically begins by using somatosensory focus to train attention control (Kerr et al., 2013). It is hypothesized that as the capacity for body awareness deepens with practice, individuals begin to have increased access to external as well as visceral and internal bodily sensations, which may facilitate earlier detection of emotional reactions or action tendencies, and therefore provide a platform for more effective self-regulation (Hölzel et al., 2011). It is hypothesized that MBIs not only focus on enhancing body awareness, but also cultivate a more accepting, non-judgmental non-reactive mode of processing interoceptive signals.

A recent meta-analysis (Treves et al., 2019) specific to studies measuring the relationship between objectively measured interoceptive accuracy (using 13 separate tasks spanning multiple parts of the body) and mindfulness found a small but significant relationship across 17 independent samples (*g* = 0.21). When studies were examined separately by trial design, only RCT studies maintained a significant relationship, although effect sizes were similar in magnitude across all study designs. A further meta-analysis of only heartbeat-related interoceptive accuracy studies (Khalsa et al., 2020), which included two studies that were not in Treves et al. (2019), found that across eight studies (12 experiments) there was no relationship between meditation and heartbeat-related interoceptive accuracy. The authors concluded that the practice of meditation is not related to improved cardiac interoception (Khalsa et al., 2020).

A few studies have also found a relationship between meditation and self-reported confidence ratings of interoceptive accuracy during behavioral tasks (Parkin et al., 2014; Fischer et al., 2017). Interestingly, in an uncontrolled study of participants who took an 8-week MBSR or MBCT course and were measured pre- and post- intervention (Parkin et al., 2014), subjective confidence in a heartbeat counting task significantly improved pre-to post-intervention while task performance did not. Khalsa et al. (2008) found a similar pattern of results through a cross-sectional study comparing long-term Vipassana and Tibetan Buddhist meditators to non-meditator controls such that differences in behavioral measures of heartbeat detection were not found, but meditators self-reported greater confidence in their ratings and lesser task difficulty than controls.

Early studies have also found mindfulness has an effect on interoception when interoception is self-reported using a questionnaire based on an expanded definition of interoception that includes cognitive and affective dimensions of the processing of bodily information (i.e., the MAIA; Mehling et al., 2018). de Jong et al. (2016) found that self-regulation and not-distracting facets of interoception increased significantly more in an MBCT condition than a TAU condition in a sample of 40 participants with chronic pain and comorbid depression. Gawande et al. (2019) similarly found that a non-traditional MBI (Mindfulness Training for Primary Care; MTPC) led to significantly greater self-reported interoception than a low-dose comparator control group. Bornemann et al. (2015) found that attention regulation, emotion awareness, self-regulation, body listening, and body trusting facets of self-reported interoception significantly increased through a 3-month contemplative intervention that emphasized body scan and breathing meditations when compared to a retest control group. Interestingly, the noticing facet, which is most representative of a narrower definition of interoception, did not significantly differ between intervention and control groups in any of these studies.

Very few studies of the relationship between interoception and meditation have included mental health outcomes in their designs. Evidence for target validation also varies by type of process and mode of measurement. Bornemann and Singer (2017) conducted a 9-month mindfulness-training intervention with two cohorts, a 3-month mindfulness-training intervention with a third cohort, and a retest control group. Significant time by condition interactions indicated that both heartbeat counting and emotional awareness improved over time more in the mindfulness group than the control group. Further, residualized changes in heartbeat counting between baseline and 3 months predicted overall residualized changes in emotional awareness from baseline to 9 months. However, given that meta-analyses have found no relationship between meditation and heart-beat detection-based interoception, these results are not representative of overall findings (Khalsa et al., 2020). Daubenmier et al. (2013) compared experienced Vipassana meditators and non-meditators on a measure of respiratory interoceptive accuracy (a respiratory discrimination task). While group differences were found in resistive load discrimination, these differences were not correlated with state or trait anxiety. Only one study examined changes in interoceptive attention as a mediator of mental health outcomes in an MBI; results from de Jong et al. (2016) showed that changes in depression were partially mediated by changes in a self-reported not-distracting facet of interoception. In sum, more research is needed to validate interoception and its numerous aspects.

#### Selflessness and Self-Transcendence

While interoception-related mechanisms suggest that an *increase* in information from self-specifying embodied processes would be related to MBIs benefits, other theories instead propose that a *decrease* in self-specifying embodied processes should lead to wellbeing. Multiple review papers acknowledge that various pre-reflective SRPs – that is, embodied SRPs that constitute the self-as-subject – can be diminished, attenuated or “transcended” in the context of meditation (Millière et al., 2018; Taves, 2020). The attenuation through meditation of embodied senses of self, including agency, ownership and boundaries between self and world, has been hypothesized to lead to greater wellbeing (Dambrun, 2016; Hadash et al., 2016).

In theoretical and preliminary empirical studies, experiences of a perceived diminishment or dissolution of the boundaries that separate self and world have been defined and measured through an array of largely overlapping constructs, including self-transcendent experience (Yaden et al., 2017), lack of sense of boundaries (Ataria, 2015; Ataria et al., 2015), non-dual awareness (Hanley et al., 2018), oneness experience (Van Lente and Hogan, 2020), and selflessness (Dor-Ziderman et al., 2013; Dambrun, 2016). Confusingly, in other contexts the identical terms selflessness (Dambrun and Ricard, 2011; Hanley et al., 2017) and self-transcendence (Vago and Silbersweig, 2012; Kang, 2019) have been used to refer to allocentric forms of identity – a social identity construct about the degree to which other people are included in one’s self-concept. This interpersonal form of self-transcendence has also often been linked to empathy and prosocial behaviors and is generally understood as a trait rather than a state. Theoretical and empirical relationships between attenuations in embodied SRPs and changes in allocentric social identity have yet to be established. Thus, at present, the multiple ways in which “selflessness” and “self-transcendence” have been operationalized appears to be a clear instance of the jingle fallacy among SRPs evaluated in meditation research.

In terms of target engagement by mindfulness training, only one study (Garland et al., 2019) investigated any of these phenomena in an RCT of a non-standard MBI. Mindfulness-Oriented Recovery Enhancement (MORE; Garland, 2013) is an 8-week program that uses mindfulness training (mindful breathing and body scan), savoring, reappraisal and “non-dual states of consciousness” to treat addiction and chronic pain (Garland et al., 2019). In an RCT for chronic pain and opioid misuse, participants in the MORE intervention had a greater increase in self-transcendence scores than the active control, a discussion support group (Garland et al., 2019).

Five studies examined the effects of meditation on the boundaries of the sense of self in the context of randomized experimental inductions with non-clinical populations (Dambrun, 2016; Hanley et al., 2018, 2020; Dambrun et al., 2019; Hanley and Garland, 2019). Eleven to 21 min mindfulness meditation inductions reduced the salience of body boundaries to a greater degree than active listening or rest control conditions (Dambrun, 2016; Dambrun et al., 2019; Hanley et al., 2020) but not more than relaxing music (Dambrun et al., 2019). Similarly, 11 min mindfulness meditation inductions increased feelings of unity and self-expansion into the world to a greater degree than active listening control conditions (Hanley et al., 2018, 2020; Hanley and Garland, 2019). Two cross-sectional studies found higher feelings of unity and self-expansion into the world in individuals with more self-reported meditation experience (Hanley et al., 2018; Hanley and Garland, 2019). Most of these studies have investigated meditation related changes in self-specifying processes on outcomes. Garland et al. (2019) found that changes in unity experiences pre- to post-intervention significantly mediated the effects of condition randomization on pain scores, explaining 22% of the variance in post-treatment pain scores.

A few experimental induction studies discussed above also included tests for whether state changes pre- to post-induction mediated the effects of condition on changes in current affect or subjective happiness pre- to post-session. Dambrun et al. (2019) found that condition-related increases in “unified consciousness” significantly mediated the effects of condition assignment (body scan vs. relaxing music vs. active listening) on state increases in current happiness. Similarly, Dambrun (2016) found that condition related state changes in the boundaries of the sense of self and self expansion into the world significantly mediated the effects of condition assignment (body scan vs. rest) on state increases in current happiness, but not anxiety. Finally, Hanley and Garland (2019) found that state changes in self-expansion into the world pre- to post-induction significantly mediated the relationships between condition assignment (mindfulness training vs. active listening) and both state positive and state negative affect, as measured by single items.

Evidence for self-transcendence or selflessness as a mechanism of MBIs is limited. A systematic review of potential MBI mechanisms was unable to find any studies which have tested these variables as mediators of MBIs (Gu et al., 2015). Instead, these constructs have been assessed in cross-sectional or brief induction studies with current affective states (rather than standard clinical mental health measures) as outcomes. Similarly, with the exception of MORE, all of the studies were conducted in healthy, non-clinical samples or meditators. In addition, none of the study designs included more than two measurement time points. Thus, without evidence for temporal precedence, these SRPs will continue to be considered co-occurring states, rather than mechanisms of change.

### Measurement of Self-Related Processes

In this evidence map review, the researchers determined that the measurement of SRPs is well defined for some constructs, whereas other constructs have limited measurement tools and extant assays for some SRPs are surrounded by controversy. As such, we provide the reader with guidance about possible ways to measure SRPs. Table 1 provides a summary of commonly used SRP measurement tools in MBIs. To our knowledge, well-validated assays exist to measure the SRPs of rumination, dysfunctional attitudes, and negative automatic thoughts. However, it should be noted that most rumination scales contain a negatively valenced self-evaluation dimension (brooding) and a neutral or non-valenced self-focus dimension (reflection). It is the negatively valenced dimension (brooding) and not reflection that is both reduced by MBIs and mediates reductions in depression (Shahar et al., 2010; Armstrong and Rimes, 2016). In contrast, MBI studies that combined the two scales often found no effect (Robins et al., 2012; Eisendrath et al., 2016). In other words, it is the negative self-evaluation dimension of rumination rather than self-focus alone that explains the beneficial effects of MBIs on depressive symptoms.

**TABLE 1:** Common measurement tools for self-related processes.

**Self-Related Process**	**Assay**	**Description**	**Sample items**
Decentering	Experiences Questionnaire (EQ)	Two subscales: Rumination (6 items) and Decentering/Wider Perspectives (14 items), which captures disidentification, non-reactivity, and dereification; 20 total items.	(Indicate frequency) Decentering ● I can slow my thinking at times of stress. Rumination ● I analyze why things turn out the way they do.
	Toronto Mindfulness Scale (TMS)	Two subscales: Curiosity (6 items) and Decentering (7 items), which captures disidentification, dereification, acceptance, and openness; 13 total items.	(Rate agreement) Decentering ● I experienced myself as separate from my changing thoughts and feelings. Curiosity ● I was curious to see what my mind was up to from moment to moment.
Dysfunctional attitudes	Dysfunctional Attitudes Scale (DAS)	Single scale measures depressogenic schemata, such as black-and-white thinking and negatively biased forms of self-appraisal; 40 items.	(Rate agreement) ● My life is wasted unless I am a success. ● If I do well, it is probably due to chance, if I do badly it is probably my own fault.
Interoceptive accuracy	13 objective behavioral tasks	Examples of measurements include: heartbeat counting, heartbeat detection, joint position, and blood glucose accuracy.	Heartbeat counting ● Estimate number of heartbeats. Heartbeat detection ● Detect heartbeat and report when it occurred relative to an external stimulus.
Interoceptive attention	Multidimensional Assessment of Interoceptive Awareness-2 (MAIA-2)	Eight subscales to assess dimensions of interoceptive attention: Attention regulation (7 items), Body listening (3 items), Emotional awareness (5 items), Noticing (4 items), Not-distracting (6 items), Not-worrying (5 items), Self-regulation (4 items), and Trusting (3 items); 37 total items.	(Indicate frequency) Attention regulation ● I can return awareness to my body if I am distracted. Body listening ● When I am upset, I take time to explore how my body feels.
Negative automatic thoughts	Automatic Thoughts Questionnaire (ATQ)	Single scale measures frequency of negative self-statements; 30 items.	(Indicate frequency of thought): ● I’m no good. ● My life is a mess.
Rumination	Ruminative Response Scale (RRS)	Single scale measures frequency of thoughts or actions in response to feeling depressed; 22 items.	(Indicate frequency of thought): ● Think about how alone you feel. ● Think “Why can’t I get going?”
	Rumination Reflection Questionnaire (RRQ)	Two subscales: Rumination (12 items) and Reflection (12 items); 24 total items.	(Rate agreement) Rumination ● I often find myself re-evaluating something I’ve done. Reflection ● I love exploring my “inner” self.
Self-compassion	Self-Compassion Scale (SCS)	Six subscales: Self-kindness (5 items), Self-judgment (5 items), Isolation (4 items), Common humanity (4 items), Mindfulness (4 items), and Over-identification (4 items), plus total composite score; 26 total items.	(Indicate frequency): ● I try to be loving toward myself when I’m feeling emotional pain. ● I try to see my failings as part of the human condition.
Self-efficacy	Generalized Self-Efficacy Scale (GSES)	Single scale measures self-beliefs related to coping with demands of life; 10 items.	(Rate how true/accurate): ● I can solve most problems if I invest the necessary effort. ● If I am in trouble, I can usually think of a solution.
	Self-Efficacy Scale (SES)	Two subscales: general self-efficacy (17 items) and social self-efficacy (6 items); 23 total items.	(Rate agreement) General ● I avoid facing difficulties (*reverse score*) Reflection ● I do not handle myself well in social gatherings.
Self-esteem	Rosenberg Self-Esteem Scale (RSES)	Single scale measures global self-competence and worthiness; 10 items.	(Rate agreement) ● At times I think I am no good at all (*reverse score*) ● I take a positive attitude toward myself.
Self-transcendence	Non-dual Awareness Dimensional Assessment (NADA)	State and Trait versions. Two subscales: Bliss (4 items), a measure of positive affect, and Self-transcendence (9 items), including items related to self-world boundaries and experiences of unity; 13 items.	(Indicate frequency) Self-transcendence ● I have had an experience in which the boundaries of my self dissolved. Bliss ● I have experienced an all-embracing love.
	The Perceived Body Boundaries Scale (PBBS)	Single item assesses the sense of being more or less separated from the world.	A series of images of human forms with increasingly transparent boundaries to indicate gradations of body boundary salience, with the intention of measuring the boundaries of the self, which may or may not align with the boundaries of the physical body.
	Spatial Frame of Reference Continuum (SFoRC)	Single item in which individuals rate how far their sense of self expands into the world.	A single image of a human form surrounded by concentric circles that expand outward. Respondents are asked to locate how far their sense of self extends into the world.

*● ATQ: Hollon and Kendall (1980).*

*● DAS: Weissman and Beck (1978).*

*● EQ: Fresco et al. (2007).*

*● GSES: Schwarzer and Jerusalem (1995).*

*● MAIA-2: Mehling et al. (2018).*

*● NADA: Hanley et al. (2018).*

*● PBBS: Dambrun (2016).*

*● RRS: Treynor et al. (2003).*

*● RRQ: Trapnell and Campbell (1999).*

*● RSES: Rosenberg (1965).*

*● SCS: Neff (2003).*

*● SES: Sherer et al. (1982).*

*● SFoRC: Hanley and Garland (2019).*

*● TMS: Lau et al. (2006).*

Additionally, validated assays exist to measure decentering, self-compassion, self-efficacy, and self-esteem. The Self-Compassion Scale (SCS; Neff, 2003) is the most widely used scale for measuring self-compassion, but it has received some criticism over its construct validity and its status as a therapeutic mechanism (Muris and Otgaar, 2020). Half of the scale is comprised of “uncompassionate self-responding” reversed items that are highly correlated with internalizing psychopathology (anxiety and depression). Thus, use of the SCS total score could obscure what processes are occurring in therapy, and inflate the relationship with psychopathology. In rebuttal, Neff acknowledged that the two sides of the scale (i.e., compassionate and uncompassionate self-responding) are distinct but related, and presented empirical data to defend the validity of the SCS (Neff, 2016, 2020). Nevertheless, a number of new self-compassion-related scales have been developed (Kraus and Sears, 2009; Gilbert et al., 2011; Gu et al., 2020), but have yet to be widely utilized in MBI research (Kraus and Sears, 2009; Gilbert et al., 2011; Gu et al., 2020).

Many mindfulness scales, including the Five Facet Mindfulness Questionnaire (FFMQ) (Baer et al., 2006) and the Freiburg Mindfulness Inventory (FMI) (Walach et al., 2006), contain items related to decentering. However, Bernstein et al. (2015) identified two assays that specifically target decentering within MBIs: the decentering subscale of the Experiences Questionnaire (EQ; Fresco et al., 2007) and the Toronto Mindfulness Scale (TMS; Lau et al., 2006), which was based on an operational definition of mindfulness by Bishop et al. (2004) related to non-elaborative attention characterized by curiosity, acceptance, and openness to experience, with all items referring to an immediately preceding meditation session. Decentering is plagued by imprecision and issues with construct validity (Bernstein et al., 2019). At least fifteen different terms are in use to refer to decentering-like processes including cognitive defusion, cognitive distancing, decentering, detached mindfulness, disidentification, metacognitive awareness, metacognitive mode, observing self reperceiving, self-as-context, self-distancing, third-person perspective, and witness consciousness (Deikman, 1982; Masuda et al., 2004; Bernstein et al., 2015). While many of these terms are used interchangeably, it is unclear to what extent the terms and their associated assays measure the same or different constructs. For example, while both the TMS and EQ measures for depression purportedly measure “decentering,” they are not correlated with each other, and appear to measure different constructs (jingle fallacy) (Bieling et al., 2012). The jangle fallacy also appears to be at play, as constructs with different names, such as mindfulness and decentering, are more highly correlated (*r* = 0.81) than constructs with the same name (Carmody et al., 2009). In addition, some items on the decentering scales, e.g., “I am better able to accept myself as I am” may better reflect self-compassion and self-acceptance,” than disidentification (Fresco et al., 2007).

The Multidimensional Assessment of Interoceptive Awareness (MAIA; Mehling et al., 2012, 2018) is one of the main self-report measures of interoceptive attention that is used in mindfulness research. Yet, other facets of interoception are less readily measured. Interoceptive accuracy has often been assessed via assessments of individuals’ experiences of various body areas or bodily functions (e.g., heartbeat counting). However, these tasks’ validity has been criticized by psychophysics experts (Brener and Ring, 2016; Ring and Brener, 2018). A number of other objective performance measures have been developed to assess interoceptive accuracy in different bodily systems, including measures of respiratory tracking, blood glucose, tactile sensitivity and detection, joint position and kinesthesia, proprioceptive drift, and arousal as measured through heartbeat and skin conductance (see Treves et al., 2019).

Three main validated measures have been developed for the assessment of self-transcendence in the context of meditation. The Non-dual Awareness Dimensional Assessment-Trait (NADA-T; Hanley et al., 2018) is a well validated self-report measure that was created through a factor analysis of preexisting mysticism and meditation scales. It contains two subscales: self-transcendence and bliss. The self-transcendence subscale includes multiple items pertaining to self-world boundaries such as “I have had an experience in which the boundaries of my self dissolved,” but also includes items that may be tracking other kinds of self-related changes, such as “I have experienced all notion of self and identity dissolve away” and “I have experienced the insight that ‘all is one.”’ Also embedded within it are measures not directly related to SRPs; the bliss subscale measures positive affect experiences, such as the item “I have experienced an all-embracing love.”

The Perceived Body Boundaries Scale (PBBS; Dambrun, 2016) is a single-item measure developed to assess “the sense of being more or less separated from the world” (p. 91). The PBBS uses a series of images of human forms with increasingly transparent boundaries to indicate gradations of body boundary salience, with the intention of measuring the boundaries of the self, which may or may not align with the boundaries of the physical body (Dambrun, 2016). Lastly, the Spatial Frame of Reference Continuum (SFoRC; Hanley and Garland, 2019) is a single-item measure through which participants rate how far their sense of self expands into the world. The scale uses a single image of a human form surrounded by concentric circles that expand outward. Similar to decentering and mindfulness, measures of self-transcendence and selflessness have issues with construct validity and jingle-jangle fallacies. Different terms and scales are being used to measure largely overlapping constructs. In addition, the identical terms “selflessness” and “self-transcendence” are used both to refer to a trait-based social identity construct about the degree to which other people are included in one’s self-concept (Figure 1, upper circle), as well as to refer to state-based embodied SRPs concerning self-world boundaries (Figure 1, lower circle).

## Discussion

### The Evidence (Gaps) for Target Engagement and Target Validation of Self-Related Processes in Mindfulness-Based Interventions

This knowledge synthesis investigated the potential role of self-related processes (SRPs) as a mechanism of change in standard mindfulness-based interventions (MBIs). A change or attenuation in selfhood is frequently advanced as a mechanism of MBIs, a perspective that comes at least in part from the substantial influence of the theory and practice of Buddhism on MBIs. However, which SRPs should be considered the target is often un- or under-specified. Contemporary research across multiple fields has demonstrated that the sense of self is grounded in multiple processes, ranging from pre-reflective embodied “self-as-subject” processes to conceptual and often valenced evaluative “self-as-object” processes. This evidence map has attempted (1) to synthesize and clarify which and in what way these processes are theorized to be mechanisms of MBIs, (2) to evaluate the current degree of evidence across the full range of SRPs, and (3) to indicate where research into SRPs require additional clarification and precision in order to navigate the jingle-jangle fallacy.

Based on the current review, extant data show that MBIs significantly engage some SRPs, regardless of MBI or population type, which confirms that some SRPs have a signal as potential mechanisms leading to beneficial health outcomes. Most of the SRPs that have been measured in MBI RCTs are conceptual senses of self or self-regulation skills. Specifically, the SRPs measured and engaged by MBIs include increased positive and decreased negative self-evaluation, as measured by rumination, negative thoughts and attitudes, self-compassion, self-esteem and self-efficacy. With support from multiple meta-analyses, rumination showed the strongest evidence as an MBI mechanism for depression, with other physical and mental health outcomes also supported. Self-compassion showed consistent target engagement but was inconsistently related to improved health outcomes. Self-efficacy and self-esteem were reliably engaged by MBIs but have not been evaluated as mechanisms. Evidence for decentering as a mechanism was mixed and undermined by inadequate construct validity and discrepancies between theory and assay. Across all RCTs, MBIs engaged SRPs more than passive, but not active controls, which suggests that engagement of SRPs is neither specific to nor greater in MBIs. Overall, positively and negatively valenced conceptual senses of self investigated through clinical and health psychology have received the most attention and have the larger evidence base for a mechanistic relationship with MBIs.

In contrast, very few, embodied SRPs have been measured in an MBI or longitudinal intervention study. Interoceptive processes were the only process with enough studies of any kind to facilitate a meta-analysis. Evidence for interoceptive processes as mechanisms of MBIs was mixed, and dependent on type of interoceptive process and method of measurement. Aside from interoception, measurement of other embodied self-specifying processes were entirely absent from standard MBI trials, and with the exception of one non-standard MBI trial, came mostly from experimental induction studies in healthy samples or qualitative case reports. In addition to the lack of data from clinical trials, evidence for attenuations in self-specifying process as mechanisms of MBIs or meditation is limited by measurement issues and lack of assessment of enduring outcomes.

### Limitations

While this evidence (gap) map is one of the first attempts to identify and evaluate a wide range of SRPs in MBIs, it also has many limitations. The current review may not be exhaustive, as SRPs are often measured as secondary outcomes and thus some important studies may not have been identified in this map. In addition, this review investigated the effects of meditation practice on SRPs without differentiating the types of meditation practices, which may impact target engagement and target validation (Cullen et al., 2021). Additionally, our review depended on the measurement tools used in extant research, which have some limitations. The use of well-validated SRP measures is imperative to advancing this field.

### Suggestions for Future Research

As one of the first reviews to integrate and evaluate evidence for SRPs as mechanisms in MBIs, many other questions still remain to be explored. Importantly, this evidence map suggests that greater attention needs to be placed on identifying, delineating and measuring specific SRPs. Theoretical models proposing changes in sense of self have often insufficiently specified SRPs, leaving ambiguity as to whether those processes are conceptual and evaluative “self-as-object” SRPs, pre-reflective and embodied “self-as-subject” SRPs, or self-regulation skills. They have also insufficiently attended to whether increases or attenuations of SRPs is the proposed mechanism of change. This evidence (gap) map found evidence that mindfulness training leads to decreases in certain conceptual senses of self (negative self-evaluation) and increases in others (positive self-evaluation) suggesting an important and often underemphasized role of valence. Although preliminary, current research also suggests that mindfulness training may lead certain embodied processes (e.g., sense of body boundaries) to decrease while others (e.g., interoception) increase, although how these changes are related to outcomes is still unknown. Attending to these differences remains crucial for both theoretical models and empirical research, as certain directions of change in specific SRPs have better evidence for target engagement and target validation than others, and researchers should not expect that a global attenuation of SRPs will be optimal in all circumstances (Britton, 2019).

Given that different meditation practices are intended to target different forms of selfhood in different ways, more attention should be paid to practice-specific effects (Dahl et al., 2015; Lutz et al., 2015). Furthermore, given that the current evidence emphasized novices in MBIs, with data from advanced Buddhist meditators being included only when data from MBIs was unavailable, it is possible that certain additional SRPs might change as a result of years of mindfulness practice rather than in the course of a typical 8-week MBI. However, given that the review focused on mechanisms of MBIs, it is unclear whether SRPs engaged through a longer commitment to mindfulness practice should be considered mechanisms of change for MBIs. Finally the social self – or the self in relation to others, or as part of a group – was only briefly discussed in the section on self-transcendence. Given that many forms of self-hood, both conceptual and embodied, can be influenced by both culture and group membership (Canby et al., 2020), the impacts of MBIs through social dimensions of self deserve more attention.

### Clinical Implications for the Delivery of Mindfulness-Based Interventions

The findings of the current review has several implications for mindfulness-based interventions. The strongest evidence for an SRP-based mechanism of MBIs’ beneficial effects comes from increasing positively valenced and/or decreasing negatively valenced conceptual senses of self. Consistent with other studies, this suggests that mindfulness-related wellbeing is better explained and achieved by self-concept enhancement than Buddhist-derived ego-quieting or multi-level SRP reduction (Gebauer et al., 2018). In order to maximize efficacy, those delivering MBIs may want to consider how self-concept enhancement can be emphasized in the curriculum.

Mindfulness-based intervention research on embodied SRPs remain understudied, especially in relation to outcomes. However, there is reason to think that reductions in embodied SRPs or global reductions in all SRPs is not the primary mechanism of MBI benefits. In a recent MBI trial, “self-disturbances” were among meditation-related adverse effects associated with enduring impairment (Britton et al., 2021). In addition, a qualitative study of meditation-related changes in sense of self found that reports of more global reductions in SRPs was associated with higher levels of functional impairment (Lindahl and Britton, 2019). Furthermore, attenuations in embodied SRPs are also associated with severe psychopathology, including psychotic disorders and dissociation (Sierra and David, 2011; Sass, 2014; Borda and Sass, 2015; Medford et al., 2016). Thus, strategies for decentering or “disidentification” from certain aspects of experience should perhaps be circumscribed to negative self-evaluation.

In addition to mental health risks, medical ethicists have identified epistemic costs and raised ethical concerns that MBIs are “metaphysically loaded with philosophical claims about the self” that may create tension with people’s core beliefs (Ratnayake and Merry, 2018 p, 567). The authors also point out that the metaphysical commitments of mindfulness practices means the claim that they are “universal” is “problematically misleading” (p. 567). MBIs can assuage these concerns, maximize benefits and minimize harms by basing intervention components and practice choices on the available evidence base rather than on “metaphysically loaded” exercises and theories that are not substantiated by the evidence base.

## Conclusion

Self-related processes are a promising area of research for investigating mechanisms of mindfulness-based interventions. However, senses of self are multiple, and many constructs remain poorly delineated or differentiated. Available assays often either assess multiple SRPs or have embedded within them additional features such as positive affect, which inflate their relationship with outcomes (Taves, 2020). Thus, researchers should exercise caution in specifying SRPs and in creating and selecting assays. Additionally, differentiating whether constructs refer to embodied self-specifying bodily process, to self-concepts, or to self-regulation skills is important because attenuations in the former have been associated with dissociation and other forms of psychopathology (Sierra and David, 2011; Sass, 2014; Borda and Sass, 2015; Medford et al., 2016). Given the complexity of SRPs, we should not expect that a global attenuation of SRPs through meditation will be beneficial across the board (Lindahl and Britton, 2019). Rather, attenuations or increases of specific SRPs may be beneficial for certain outcomes but not others. Maximum benefit is likely to occur when the direction of SRP engagement corrects a condition-specific deficit or excess that is a known concomitant or risk factor. Conversely, benefit is least likely when the direction of target engagement exacerbates a baseline imbalance (Britton, 2019). Current evidence does not support the theoretical claim that mindfulness training produces its salutary effects through a global and comprehensive attenuation of all forms of selfhood. Instead, this evidence map demonstrates that increases in certain conceptual SRPs and decreases in others show promise as mediators of outcomes in MBIs. However, many SRPs remain underinvestigated, requiring further research. Until then, theoretical models of and summarizing statements about the mechanisms of MBIs should identify specific SRPs and not overstate claims beyond the existing evidence.

## Author Contributions

WB, GD, SP, DV, SL, ZS-O, RF, and EM conceptualized, designed, and developed the protocol for this study. SP, WB, and JRL wrote the initial drafts. TD, JL, HK, LS, HR, AC, PA, and JS conducted literature searches, compiled data, and aided writing efforts. WB, RA, JRL, NC, DV, and EM completed the final evidence map review and writing. All authors contributed to the article and approved the submitted version.

## Conflict of Interest

WB is a MBSR and MBCT teacher and has received financial compensation for this role. WB is nominally affiliated with the Mindfulness Center at Brown University which generates income by offering mindfulness classes to the public. WB is the founder of Cheetah House, a RI non-profit organization that provides information about meditation-related difficulties, individual consultations, and support groups, as well as educational trainings to meditation teachers, clinicians, educators and mindfulness providers. JL’s spouse is the founder of Cheetah House. JL has also received compensation for providing educational trainings on meditation-related difficulties. These interests have been disclosed to and is being managed by Brown University, in accordance with its Conflict of Interest and Conflict of Commitment policies. The remaining authors declare that the research was conducted in the absence of any commercial or financial relationships that could be construed as a potential conflict of interest.

## Publisher’s Note

All claims expressed in this article are solely those of the authors and do not necessarily represent those of their affiliated organizations, or those of the publisher, the editors and the reviewers. Any product that may be evaluated in this article, or claim that may be made by its manufacturer, is not guaranteed or endorsed by the publisher.
