# Supplementary material for: From Self-Esteem to Selflessness: An Evidence (Gap) Map of Self-Related Processes as Mechanisms of Mindfulness-Based Interventions
Source: Front Psychol. 2021 Nov 22;12:730972. doi: 10.3389/fpsyg.2021.730972 (PMC8645694; doi:10.3389/fpsyg.2021.730972)
Supplement: Supplementary file 1 [file Table_1.docx]

**SUPPLEMENTAL TABLE 1.**

From Self-Esteem to Selflessness: An Evidence (Gap) Map of Self-Related Processes as Mechanisms of Mindfulness-Based Interventions

| **study** | **SRP type** | **domain** | **construct** | **assay name** | **N (k)** | **design** | **intervention(s)** | **control** | **population description** |
| --- | --- | --- | --- | --- | --- | --- | --- | --- | --- |
| Alsubaie et al. (2017) | C | Negative Self-Evaluation | Rumination, Dysfunctional Attitudes | Variety of rumination assays, DAS | n=688 (k=5) | SR | MBCT, MBSR | mix of inactive and active | Physical and psychological problems |
| Armstrong & Rimes (2016) | C | Negative/Positive Self-Evaluation | Rumination, Self-compassion | RRQ, SCS | n=34 | MBI RCT | MBCT | Online self-help course | Adults with elevated neuroticism |
| Bieling et al. (2012) | SR | Self-regulation skills | Decentering | EQ-D 14-item; TMS-D | n=84 | MBI RCT | MBCT | MBCT +ADM taper, ADM, taper + placebo | remitted depressed |
| Bornemann & Singer (2017) | E | Interoception | Heart-beat related interoceptive accuracy | Heartbeat Tracking | n=318 | mMBI RCT | 9-month mental training study (Presence, Affect, Perspective; 2 cohorts) with a third cohort only doing three months (Affect), the ReSource Project | Retest control group | Adults from community with good psychological and physical health, no intensive experience with meditation, and between ages of 20 and 55 |
| Bornemann et al. (2015) | E | interoception | Interoceptive awareness | MAIA | n=232 | mMBI RCT | 3-month contemplative intervention in the context of the ReSource project, involving daily “Body Scan” and “Breath Meditation” practices | Retest control group | Adults from community with good psychological and physical health, no intensive experience with meditation, and between ages of 20 and 55 |
| Carmody et al. (2009) | SR | Self-regulation skills | Decentering | EQ-D 11 -item | n=309 | pre-post (non-RCT) | MBSR | none | heterogeneous community sample |
| Dambrun (2016) | E | Selflessness/self-transcendence | Selflessness | Perceived Body Boundaries Scale (PBBS) | n=53 | EXI | 21 minute Body Scan | 21 minute rest condition | Psychology undergraduates |
| Dambrun et al. (2019) | E | Selflessness/self-transcendence | Selflessness/Oneness | Six oneness and timelessness/spacelessness items from the Francis and Louden scale of mystical orientation; Perceived Body Boundary Scale (PBBS) | n=89 | EXI | 21 minute Body Scan | 21 minute relaxing music and active listening to a story conditions | Psychology undergraduates |
| Daubenmier et al. (2013) | E | Interoception | Respiratory interoceptive accuracy | Respiratory Discrimination Task | n=34 | Cs | n/a | healthy adult non-meditators | Vipassana meditation practitioners with at least 5 years experience and current daily practice (at least 30 min 5x a week) |
| de Jong et al. (2016) | E | Interoception | Interoceptive awareness | MAIA (only 5/8 subscales: Noticing, Not- Distracting, Attention Regulation, Emotional Awareness, and Self-Regulation) | n=31 | MBI RCT | MBCT | TAU (waitlist + regular visits) | Adults with Chronic Pain and Comorbid Depression |
| DiRenzo et al. (2018) | SR | Self-regulation skills | Self-efficacy | Rheumatoid arthritis self-efficacy | n=73 (k=1) | MA | MBCT, MBSR, Vitality Training Program | Mixed controls | Rheumatoid Arthritis |
| Docteur et al. (2020) | C | Negative Self-Evaluation | Dysfunctional Attitudes | DAS | n=99 | MBI RCT | MBCT | Waitlist | Bipolar I |
| Ebrahimi et al. (2019) | C | Negative Self-Evaluation | Dysfunctional Attitudes | DAS | n=30 | MBI RCT | MBCT | Waitlist | infertile women |
| Eisendrath et al. (2016) | C | Negative/Positive Self-Evaluation | Rumination, Self-compassion | RRS, SCS | n=173 | mMBI RCT | modified MBCT | Health Enhancement Program | Treatment resistant depression |
| Emerson et al. (2017) | SR | Self-regulation skills | Self-efficacy | Teacher self-efficacy assays | n=209 (k=5) | SR | Any MBI with multiple sessions | Mixed controls | Teachers of 5-18 yr olds |
| Farb et al. (2018) | SR | Self-regulation skills | Decentering | EQ-D 14 item | n=166 | MBI RCT | MBCT | CBT | remitted depressed |
| Fischer et al. (2017) | E | Interoception | Self-reported confidence ratings of interoceptive accuracy | 10-point confidence rating of heartbeat perception task | n=49 | mMBI RCT | 8 weeks of listening to 20 minute body scan audiotape | audio-book control | healthy college students |
| Garland et al. (2019) | E | Selflessness/self-transcendence | Non-dual awareness | NADA (non-dual awareness dimensional assessment) | n=95 | mMBI RCT | Mindfulness Oriented Recovery Enhancement (MORE), an 8-week program that uses mindfulness training, savoring, reappraisal and “non-dual states of consciousness” to treat addiction and chronic pain | Discussion support group | Adults with chronic pain and opioid misuse |
| Gawande et al. (2019) | E | Interoception | Interoceptive awareness | MAIA | n=136 | mMBI RCT | Mindfulness Training for Primary Care (MTPC) is an 8-week, referral-based, insurance-reimbursable program integrated into safety-net health system patient-centered medical homes | A low-dose comparator (LDC) consisting of a 60-min mindfulness introduction, referral to community and digital resources, and addition to a 6-month waitlist for MTPC | Adults with DSM-IV diagnosis receiving primary care |
| Gayner et al. (2012) | SR | Self-regulation skills | Decentering | TMS-D | n=117 | MBI RCT | MBSR | TAU | HIV+ gay men |
| Golden et al. (2021) | C | Positive Self-Evaluation | Self-compassion | SCS | n=598 (k=26) | MA | MBSR/MBCT | mixed | non-clinical |
| Gu et al. (2015) | C | Negative Self-Evaluation | Rumination | Variety of rumination assays | k=6 | MA | MBCT, MBSR | mix of inactive and active | Broad variety of samples |
| Hanley & Garland (2019) | E | Selflessness/self-transcendence | Self-Transcendent Experience | Spatial Frames of Reference Continuum (SFoRC) | n=55 | EXI | 11-minute mindfulness training condition consisting of both focused attention and open monitoring | 11-minute active listening control condition | College students |
| Hanley et al. (2018) |  | Selflessness/self-transcendence | Non-dual awareness | NADA-S (non-dual awareness dimensional assessment-State) | n=53 | EXI | 11-minute standardized body scan meditation | 11-minute active listening control condition | Non-clinical participants recruited from a university setting |
| Hanley et al. (2020) | E | Selflessness/self-transcendence | Self-Transcendent Experience | Perceived Body Boundaries Scale (PBBS); Spatial Frames of Reference Continuum (SFoRC) | n=45 | EXI | 11-minute mindful breathing and body scan condition | 11-minute active listening condition | Non-clinical participants recruited from a university setting |
| Hasanzade & Khalatbari (2017) | C | Negative Self-Evaluation | Dysfunctional Attitudes | DAS | n=30 | MBI RCT | MBCT | Waitlist | hypertension |
| Hofheinz et al. (2020) | C | Negative Self-Evaluation | Automatic Thoughts | ATQ | n=72 | mMBI RCT | 6-week MBI | Cognitive intervention | Depressed adults |
| Hoge et al. (2015) | SR | Self-regulation skills | Decentering | EQ-D 11 -item | n=38 | MBI RCT | MBSR | Stress Management Education (SME). | Generalized anxiety Disorder |
| Jazaieri et al. (2012) | C | Positive Self-Evaluation | Self-esteem | RSES | n=56 | MBI RCT | MBSR | Aerobic Exercise | Adults with social anxiety disorder |
| Jermann et al. (2013) | C | Negative Self-Evaluation | Dysfunctional Attitudes; Rumination | DAS, RRQ | n=60 | MBI RCT | MBCT | TAU | Individuals with ≥3 previous depressive episodes |
| Kaviani et al (2012) | C | Negative Self-Evaluation | Dysfunctional Attitudes, Automatic Thoughts | DAS | n=30 | MBI RCT | MBCT | Waitlist | depressed students |
| Khalsa et al. (2008) | E | Interoception | Self-reported confidence ratings of interoceptive accuracy | self-reported accuracy and difficulty ratings of heartbeat detection task | n=47 | Cs | n/a | non-meditators | Kundalini meditators (N=17) and Tibetan meditators (N=13) who fit the following criteria: (1) a minimum of 15 years of formal meditation practice, (2) a self-reported strong daily practice, and (3) having attended at least one meditation retreat during the previous year |
| Khalsa et al. (2020) | E | Interoception | Heart-beat related interoceptive accuracy | 4 separate tasks measuring heart-beat related interoceptive accuracy | n=724 (k=12) | MA | Included both cross-sectional (k=5) and longitudinal (k=7), with various interventions | non-meditators (k=12) | Varied based on study (longitudinal included novice meditators, cross-sectional included long-term meditators from various traditions) |
| Khoury et al. (2013) | C | Positive Self-Evaluation | Self-compassion | SCS | n=2668 (k=29) | MA | MBSR | mix of active, inactive, uncontrolled | clinical and non-clinical samples |
| Kuehner et al. (2009) | C | Negative Self-Evaluation | Dysfunctional Attitudes | DAS | n=60 | EXI | No | 3 induction groups | Students |
| Kuyken et al. (2010) | C | Positive Self-Evaluation | Self-compassion | SCS | n=123 | RCT | MBCT | treatment as usual | depression remission |
| McConville et al. (2017) | SR | Self-regulation skills | Self-efficacy | Self-efficacy Scale | n=151 (k=2) | SR | Unknown "mindfulness programs" | Mixed controls | Mixed samples |
| Mehdipour et al (2017) | C | Negative Self-Evaluation | Dysfunctional Attitudes, Automatic Thoughts | DAS, ATQ | n=30 | MBI RCT | MBCT | Not reported | Cancer patients |
| Parkin et al. (2014) | E | Interoception | Self-reported confidence ratings of interoceptive accuracy | 100-point confidence rating of heartbeat mental tracking task | n=19 | MBI RCT (but no control condition) | MBSR and MBCT | no control | Individuals who self-referred to MBSR or MBCT course |
| Perestelo-Perez et al. (2017) | C | Negative Self-Evaluation | Rumination | Variety of rumination assays | n=781 (k=11) | MA | MBCT | mix of inactive, active, and uncontrolled | current and/or past depression |
| Perez-Blasco et al. (2013) | SR | Self-regulation skills | Self-efficacy | Maternal self-efficacy | n=26 | mMBI RCT | 8-session MBSR variation | Waitlist | breastfeeding mothers |
| Ramel et al. (2004) | C | Negative Self-Evaluation | Dysfunctional Attitudes | DAS | n=27 | MBI RCT | MBSR | Waitlist | Depression (lifetime) |
| Randal et al. (2015) | C | Positive Self-Evaluation | Self-esteem | RSES | k=32 | SR | k=6 MBSR, k=3 modified MBSR, k=1 MBCT | not specified | mixed mental/physical health |
| Robins et al. (2012) | C | Negative/Positive Self-Evaluation | Rumination, Self-compassion | RRS, SCS | n=56 | MBI RCT | MBSR | Waitlist | Healthy individuals |
| Schumer et al. (2018) | C | Negative Self-Evaluation | Rumination | Variety of rumination assays | n=5489 (k=65) | MA | Brief MBIs | mix of inactive, active, and uncontrolled | Broad variety of samples |
| Segal et al. (2019) | SR | Self-regulation skills | Decentering | decentering variable made of mindfulness, self-compassion and well-being scales | n=166 | MBI RCT | MBCT | CBT | remitted depressed |
| Shahar et al. (2010) | C | Negative Self-Evaluation | Rumination | RRS | n=52 | MBI RCT | MBCT | Waitlist | Major Depressive Disorder in partial remission |
| Treves et al. (2019) | E | Interoception | Interoceptive accuracy (all objective measures) | 13 separate tasks using objective measures | n=879 (k=17) | MA | Included trait mindfulness (k=2), long-term meditators (k=8), and RCTs with various interventions (k=7) | k=4 RCT with active control; k=3 RCT with inactive control; k=8 cross sectional with meditation-naive controls | Varied based on study |
| Turner et al. (2016) | SR | Self-regulation skills | Self-efficacy | Pain Self-efficacy Questionnaire | n=342 | MBI RCT | MBSR | CBT and usual care | chronic low back pain |
| van Dam et al. (2014) | C | Positive Self-Evaluation | Self-compassion | SCS | n=94 | MBI RCT | Modified MBSR (with some MBCT components) | waitlist | pre-clinical anxiety and depression |
| Wasson et al. (2020) | C | Positive Self-Evaluation | Self-compassion | SCS | n=1020 (k=29) | MA | MBSR | inactive or waitlist | adults/varied |
| Wilson et al. (2019) | C | Positive Self-Evaluation | Self-compassion | SCS | n=1172 (k=22) | MA | self-compassion-related therapies(k=13 includes MBSR/MBCT) | k=11 active control; k=11 waitlist control | adults/varied |
| Zarenejad et al. (2020) | SR | Self-regulation skills | Self-efficacy | Self-efficacy in coping with childbirth scale | n=70 | mMBI RCT | 6-session MBSR variation | Unknown | Pregnant women |

*SRP type*: C=conceptual; E=embodied; SR=self-regulation.

*Assay name*: ATQ=Automatic Thoughts Questionnaire; DAS=Dysfunctional Attitudes Scale; EQ-D=Experiences Questionnaire-Decentering; MAIA=Multidimensional Assessment of Interoceptive Awareness; RRS=Ruminative Response Scale; RRQ=Rumination Reflection Questionnaire; RSES=Rosenberg Self-Esteem Scale; SCS=Self-Compassion Scale; TMS-D=Toronto Mindfulness Scale-Decentering.

*Design*: Cs=Cross-sectional; EXI=Experimental induction; MA=Meta-analysis; MBI RCT=Randomized controlled trial using standard MBI; mMBI RCT=Randomized controlled trial using modified MBI; SR=Systematic review.
