# Supplementary material for: From Self-Esteem to Selflessness: An Evidence (Gap) Map of Self-Related Processes as Mechanisms of Mindfulness-Based Interventions
Source: Front Psychol. 2021 Nov 22;12:730972. doi: 10.3389/fpsyg.2021.730972 (PMC8645694; doi:10.3389/fpsyg.2021.730972)
Supplement: Supplementary file 2 [file Data_Sheet_1.docx]

**SUPPLEMENTAL METHODS**

From Self-Esteem to Selflessness: An Evidence (Gap) Map of Self-Related Processes as Mechanisms of Mindfulness-Based Interventions

**Introduction**

The review of self-related processes (SRPs) was part of larger review that examined three types of processes engaged by Mindfulness-Based Interventions (MBIs): cognitive processes, emotion-related processes, and self-related processes. The protocol, search, screening and data collection for all three domains were conducted at the same time, while data synthesis and analysis were conducted separately, according to the results generated for that domain. The cognitive and emotion-related processes reviews are published (Hoge et al., 2021; Whitfield et al., 2021) and the sections below describe the combined and specific methods for the review and subsequent evidence map of self-related processes.

**Systematic Review**

We initiated and anchored the evidence map with a systematic review of target engagement in RCTs of standard MBIs since they present the strongest evidence for causal inference of the effects of mindfulness meditation training on specific outcomes.

**Methods**

Established methodologies outlined by the Cochrane Handbook of Systematic Reviews (Higgins et al., 2019) and the Agency for Healthcare Research and Quality’s Methods Guide for Comparative Effectiveness Reviews (AHRQ, 2014) were used to conduct this review. The Preferred Reporting Items for Systematic Reviews and Meta-Analyses (PRISMA) guidelines were followed for reporting (Liberati et al., 2009).

***Protocol and registration***

On 12/09/16, a PROSPERO review registration for the protocol for this systematic review was submitted. The review documents were reviewed and documentation was processed on 12/16/16 (PROSPERO 2016:CRD42016051765). The posted protocol is available online from www.crd.york.ac.uk/PROSPERO/display_record.asp?ID=CRD42016051765.

***Eligibility Criteria***

Eligible studies were English-language RCTs of standard Mindfulness-Based Cognitive Therapy (MBCT), Mindfulness-Based Stress Reduction (MBSR), or variations thereof (within the parameters described below) with one or more active and/or inactive control condition(s) were included. Only studies with a sample size of at least 10 participants in the MBI arm were included, with all participants 18 years old or older.

To be considered a standardized MBSR or MBCT intervention, the curriculum must have been delivered in person, in a group setting, over eight weeks, in 1.5 to 3 hours-long weekly classes and with an accompanying all-day (~7-hour) retreat, following the respective manuals for these interventions (Kabat-Zinn, 2013; Segal, Williams, & Teasdale, 2013). Interventions delivered individually were excluded because the group process (including discussion, inquiry, and dyads) is an important component of the curriculum that takes up a large proportion of the class content.

In addition to standardized MBSR and MBCT, we also included variations of these interventions that met the following criteria: (a) total class/session time, including retreat, was at least 15 contact hours, i.e., 75% of the 20 hours typically prescribed in MBSR or MBCT; (b) the MBI included a retreat day of any duration; (c) the MBI was delivered in an in-person, group-based format; and, (d) it could include sample-specific tailoring or exercises or psychoeducation that were specific to the targeted condition (e.g., smoking cessation), as long as at least 50% of the original MBSR or MBCT content was retained.

***Information sources and search***

In addition to search terms beginning with or including the word “self”, an expert panel of five mindfulness researchers generated 30 SRP constructs to serve as search terms, namely: *agency, body awareness, decentering, disidentification, ego, embodiment, experiential self, identity/identification, interoception, meta-awareness, narrative self, observing self, perceived control, reperceiving, self-awareness, self-compassion, self-criticism, self-distancing, self-efficacy, self-esteem, self-loathing, self-monitoring, self-praise, self-referential processing, self-regulation, self-related rumination, self-worth, sense of agency, sense of control,* and *sense of ownership*.

Studies published through July 31, 2016, were included in the original search with no constraint on the starting date of publications. The AND operator was used to connect terms designed to find mindfulness studies to terms that relate to the domains of self-regulation. The Medical Subject Heading (MeSH) terms of Mindfulness and Meditation were used in addition to the terms related to emotion regulation. Terms were truncated with an asterisk (*) to capture all possible endings for the designated root of the specified word. Filters were used to restrict the retrieved citations to English-only, human-only, and adult-only (age over 18 years old) publications.

Searches were performed in the electronic databases of PubMed, PsycINFO, CINAHL (Cumulative Index to Nursing and Allied Health Literature), and ERIC (Education Resources Information Center). We supplemented our review with grey literature searches. We searched the Open Grey (http://www.opengrey.eu/) and New York Academy of Medicine Grey Literature Report (http://www.greylit.org/) electronic databases, the American Mindfulness Research Association (AMRA) ClinicalTrials.gov; PubMed Similar Articles; and ISI Web of Science back references;

***Excluded Measures***

Of note, two types of measures were excluded from this systematic review: neuroimaging measures and mindfulness scales. Neuroimaging measures were excluded to prevent redundancy with several existing neuroimaging reviews focused on mindfulness and meditation (Boccia, Piccardi, & Guariglia, 2015; Fox et al., 2016; Gotink, Meijboom, Vernooij, Smits, & Hunink, 2016; Sperduti, Martinelli, & Piolino, 2012; Tomasino, Fregona, Skrap, & Fabbro, 2012). In addition, neuroimaging data are qualitatively distinct and thus not easily combined with other data types such as questionnaires. Mindfulness self-report scales were excluded because several reviews on mindfulness scales have been published in recent years (Alsubaie et al., 2017b; Bergomi, Strohle, Michalak, Funke, & Berking, 2013; Gu, Strauss, Bond, & Cavanagh, 2015; Park, 2013; Quaglia, Braun, Freeman, McDaniel, & Brown, 2016; Sauer, Ziegler, Danay, Ives, & Kohls, 2013; Siegling & Petrides, 2014; Visted, Vollestad, Nielsen, & Nielsen, 2015). In addition, as argued by Bernstein, Hadash, and Fresco (2019), SRPs do not appear to be well measured by the currently available mindfulness scales

***Study selection***

The removal of duplicate citations was performed first using EndNote (version X7.7.1) and was then manually completed by two of the Master’s level reviewers. Titles and abstracts were double-screened using the Abstrackr software (Wallace, Small, Brodley, Lau, & Trikalinos, 2012). Each citation was randomly allocated to two reviewers who independently rated each title and abstract as *yes* (i.e., full text to be reviewed), *no* (to exclude), or *maybe* (i.e., expert opinion needed). Each citation for which both reviewers did not unanimously agree on the inclusion or exclusion status was discussed among three graduate students and the principal investigator. The final decision on inclusion or exclusion status was made by the principal investigator. After title and abstract screening, all citations were further reviewed in detail for eligibility.

***Data collection***

The publicly available Systematic Review Data Repository (SRDR) online system (http://srdr.ahrq.gov) was used to by 13 pairs of extractor-reviewers to extract the following data from the selected full-text publications: population sample characteristics; descriptions of the interventions (MBI and control(s), exposures) and comparators analyzed; outcome definitions; sample sizes; study design features, including assessment time points; target engagement results; and risk of bias. Mean extractor-reviewer agreement was 94.3% (range = 86.4% − 100%). Overall inter-rater agreement was very high (kappa = .933; range = .851 − .990).

**Results of initial systematic review**

Of the 10,802 studies identified and screened, only 14 standard MBI RCTs assessed SRPs. In addition, only five of the 30 SRP constructs generated by the expert panel had been measured within an RCT of a standard MBI, namely: self-evaluation, self-compassion, rumination, self-esteem, and self-efficacy.

**
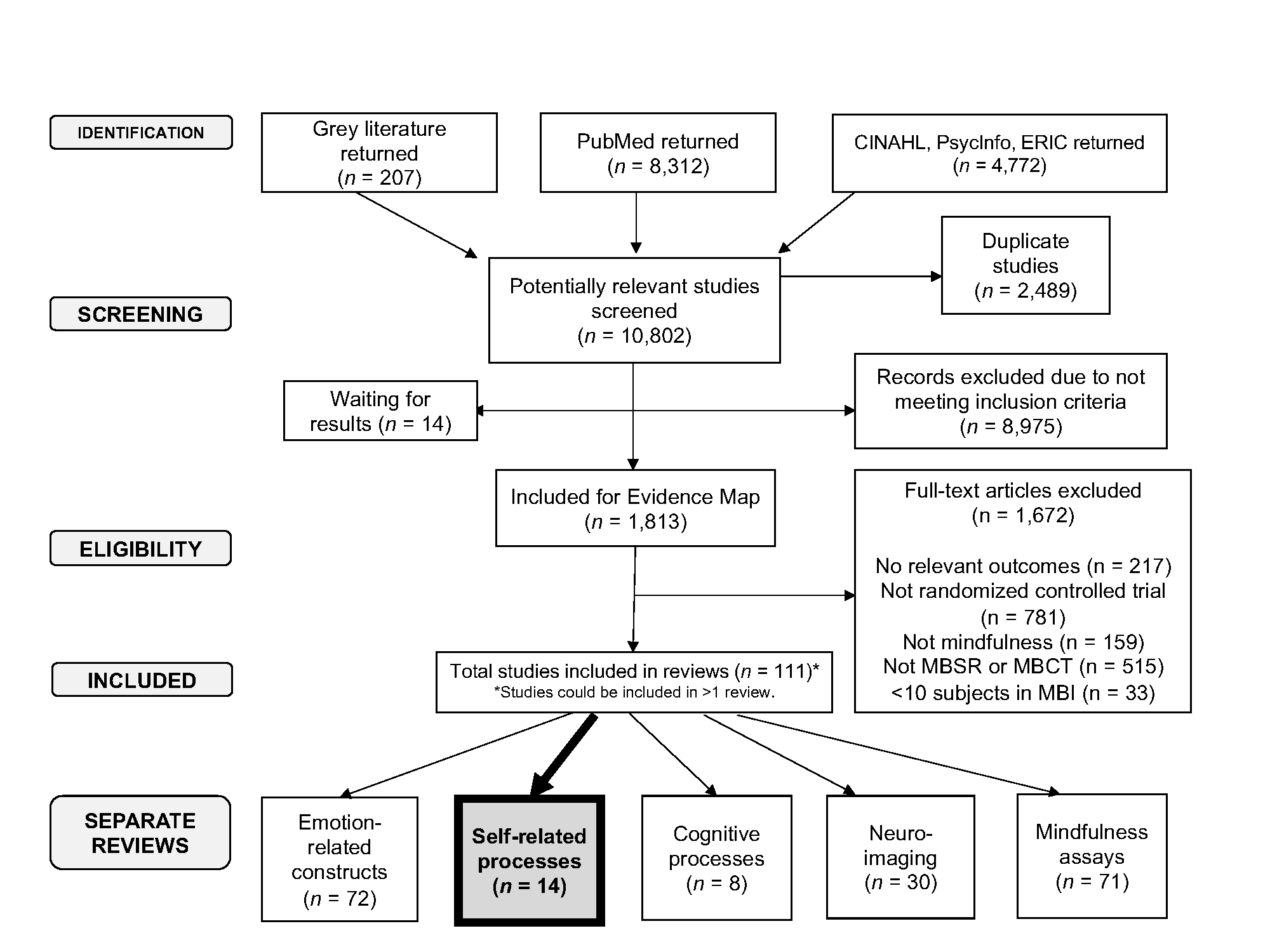
**

**Figure 1 PRIMSA Flowchart**

**Experimental Evidence (Gap) Map**

Because the initial review demonstrated that very few SRPs had been measured in the context of standard MBI RCTs, we adapted our mapping approach to match the inchoate state of the research. The team agreed to specifically include and emphasize (and allow less rigorous inclusion criteria for) SRP constructs that have been theorized as central or key mechanism of MBIs and/or represent self-as-subject SRPs. Specifically, in addition to the 5 constructs produced in the initial review, the team voted to include *interoception, decentering, selflessness and self-transcendence*, which are theorized but understudied mechanisms of MBIs (Hölzel et al., 2011; Vago & David, 2012).

The second round of reviews with modified methods was undertaken to represent SRP constructs that have been assessed in standard MBI RCTs since our last search, and/or with less rigorous designs, including modified MBIs, inductions, and cross-sectional studies. To assess engagement in MBIs in the most up-to-date research, we supplemented the first review with an integrated meta-review that was performed in January 2021, searching for the highest form of evidence for each SRP (e.g., meta-analyses), and then systematically working down the evidence ladder as needed, for lesser studied or key SRPs.

The modified approach included reviewing SRPs as mechanisms of MBIs according to the experimental medicine approach (Riddle & Science of Behavior Change Working Group, 2015). Specifically, each construct included five parts:

1) ***Target identification***

Target identification included a description of the construct and how it is theorized to function in MBIs, specifically if SRP target is theorized to increase or decrease with mindfulness exposure Constructs that had many names or the same name is used for different constructs (jingle-jangle were discussed in commentary section.

2*)* ***Assays or measures***

Assay descriptions included type of measure (self-report, behavior task etc.), and validation information, if available. Conversely, if there were problems or limitations with the assay, for example it doesn’t accurately reflect the theorized construct, or has other problems, this was discussed in the commentary and/or measurement section

3) ***Target engagement***

Target engagement refers to the degree to which the target measure is modified or changed by mindfulness exposure. Descriptions of target engagement by mindfulness meditation training are reviewed according to a hierarchical levels of evidence. The highest level of evidence were meta-analyses of MBI RCTs where an SRP fully mediated desired outcomes (Kazdin, 2007) although few, if any, SRPs met this criteria (Alsubaie et al., 2017a; Gu et al., 2015; van der Velden et al., 2015). If no meta-analyses of MBI RCTs were available, then single RCTs of standard, then modified MBIs were described. If the target had not been tested in a longitudinal RCT at all, then brief experimental meditation induction studies were permitted. If induction studies were absent, then cross-sectional studies of meditators vs non-meditators were reviewed. Finally, for some SRPs with very little empirical research, single cases, or case series of phenomenological interviews were included as the lowest level of evidence (meta-analysis of MBI RCTs > MBI RCTs > modified MBI RCTs > induction studies > cross-sectional studies > case studies or qualitative interviews);

The following PICOs were extracted from each study:

**P**articipants (clinical, non-clinical; and if clinical type (anxiety, cancer etc.). If meditators, describe selection criteria.

**I**ntervention. Original review was standard 8 week MBSR or MBCT. Describe if modified MBIs or related intervention. If experimental induction, describe practice and duration. If no intervention (meditation practice), but just cross-sectional study of meditators vs non-meditators, state.

**C**ontrol- active, passive, established treatment?

**O**utcomes- describe target engagement in detail. Assay total vs subscale? Report ES (if available or NR if not reported). Specify whether ES refers to pre-post or compared to control. Report if qualified (subgroup analysis)

***3) Target validation***

Target validation refers to the degree to which target engagement account for desired outcomes, using similar levels of evidence above, with Kazdin’s full mediation criteria at the top (Kazdin, 2007). The highest level of evidence for a mechanism is a full meditation, which requires the intervention to significantly engage (increase/decrease) the target, and that target engagement occurs first and subsequently predicts outcomes over and above the direct effect of the intervention on outcome. See Gu et al, (2015) and Kazdin (2007) for more detailed description).

***4) Commentary***

A commentary section was used to discuss caveats and other limitations of each construct and measure, including recommendations for research.

**References**

AHRQ. (2014). Methods Guide for Effectiveness and Comparative Effectiveness Reviews. In. Rockville, MD: Agency for Healthcare Research and Quality.

Alsubaie, M., Abbott, R., Dunn, B., Dickens, C., Keil, T. F., Henley, W., & Kuyken, W. (2017a). Mechanisms of action in mindfulness-based cognitive therapy (MBCT) and mindfulness- based stress reduction (MBSR) in people with physical and/or psychological conditions: A systematic review. *Clin Psychol Rev, 55*, 74-91. doi:10.1016/j.cpr.2017.04.008

Alsubaie, M., Abbott, R., Dunn, B., Dickens, C., Keil, T. F., Henley, W., & Kuyken, W. (2017b). Mechanisms of action in mindfulness-based cognitive therapy (MBCT) and mindfulness-based stress reduction (MBSR) in people with physical and/or psychological conditions: a systematic review. *Clinical Psychology Review, 55*, 74-91. doi:10.1016/j.cpr.2017.04.008

Bergomi, C., Strohle, G., Michalak, J., Funke, F., & Berking, M. (2013). Facing the dreaded: does mindfulness facilitate coping with distressing experiences? A moderator analysis. *Cognitive Behaviour Therapy, 42*(1), 21-30. doi:10.1080/16506073.2012.713391

Bernstein, A., Hadash, Y., & Fresco, D. M. (2019). Metacognitive processes model of decentering: emerging methods and insights. *Current Opinion in Psychology, 28*, 245-251. doi:10.1016/j.copsyc.2019.01.019

Boccia, M., Piccardi, L., & Guariglia, P. (2015). The meditative mind: a comprehensive meta-analysis of MRI studies. *Biomed Res Int, 2015*, 419808. doi:10.1155/2015/419808

Fox, K. C. R., Dixon, M. L., Nijeboer, S., Girn, M., Floman, J. L., Lifshitz, M., . . . Christoff, K. (2016). Functional neuroanatomy of meditation: A review and meta-analysis of 78 functional neuroimaging investigations. *Neuroscience and Biobehavioral Reviews, 65*, 208-228. doi:10.1016/j.neubiorev.2016.03.021

Gotink, R. A., Meijboom, R., Vernooij, M. W., Smits, M., & Hunink, M. G. (2016). 8-week Mindfulness Based Stress Reduction induces brain changes similar to traditional long-term meditation practice - A systematic review. *Brain Cogn, 108*, 32-41. doi:10.1016/j.bandc.2016.07.001

Gu, J., Strauss, C., Bond, R., & Cavanagh, K. (2015). How do mindfulness-based cognitive therapy and mindfulness-based stress reduction improve mental health and wellbeing? A systematic review and meta-analysis of mediation studies. *Clin Psychol Rev, 37*, 1-12. doi:10.1016/j.cpr.2015.01.006

Higgins, J. P. T., Thomas, J., Chandler, J., Cumpston, M., Li, T., Page, M. J., & Welch, V. A. (2019). *Cochrane Handbook for Systematic Reviews of Interventions version 6.0 (updated August 2019). Available from* [*www.training.cochrane.org/handbook*](www.training.cochrane.org/handbook)*.* (6.0 ed.): Cochrane.

Hoge, E. A., Acabchuk, R. L., Kimmel, H., Moitra, E., Britton, W. B., Dumais, T., . . . Fulwiler, C. (2021). Emotion-related constructs engaged by mindfulness-based interventions: A systematic review and meta-analysis. *Mindfulness (N Y), 12*(5), 1041-1062. doi:10.1007/s12671-020-01561-w

Hölzel, B. K., Lazar, S. W., Gard, T., Schuman-Olivier, Z., Vago, D. R., & Ott, U. (2011). How Does Mindfulness Meditation Work? Proposing Mechanisms of Action From a Conceptual and Neural Perspective. *Perspect Psychol Sci, 6*(6), 537-559. doi:10.1177/1745691611419671

Kabat-Zinn, J. (2013). *Full catastrophe living: Using the wisdom of your body and mind to face stress, pain, and illness*. New York, NY: Bantam.

Kazdin, A. E. (2007). Mediators and mechanisms of change in psychotherapy research. *Annu Rev Clin Psychol, 3*, 1-27. doi:10.1146/annurev.clinpsy.3.022806.091432

Liberati, A., Altman, D. G., Tetzlaff, J., Mulrow, C., Gotzsche, P. C., Ioannidis, J. P. A., . . . Moher, D. (2009). The PRISMA Statement for Reporting Systematic Reviews and Meta-Analyses of Studies That Evaluate Health Care Interventions: Explanation and Elaboration. *Plos Medicine, 6*(7). doi:10.1371/journal.pmed.1000100

Park, C. (2013). Mind-body CAM interventions: current status and considerations for integration into clinical health psychology. *J Clin Psychol, 69*(1), 45-63. doi:10.1002/jclp.21910

Quaglia, J. T., Braun, S. E., Freeman, S. P., McDaniel, M. A., & Brown, K. W. (2016). Meta-analytic evidence for effects of mindfulness training on dimensions of self-reported dispositional mindfulness. *Psychol Assess, 28*(7), 803-818. doi:10.1037/pas0000268

Riddle, M., & Science of Behavior Change Working Group. (2015). News from the NIH: using an experimental medicine approach to facilitate translational research. *Transl Behav Med, 5*(4), 486-488. doi:10.1007/s13142-015-0333-0

Sauer, S., Ziegler, M., Danay, E., Ives, J., & Kohls, N. (2013). Specific objectivity of mindfulness—A Rasch analysis of the Freiburg Mindfulness Inventory. *Mindfulness, 4*(1), 45-54. doi:10.1007/s12671-012-0145-y

Segal, Z. V., Williams, J. M., & Teasdale, J. D. (2013). *Mindfulness-based cognitive therapy for depression* (2nd ed.). New York, NY: Guilford Press.

Siegling, A. B., & Petrides, K. V. (2014). Measures of trait mindfulness: Convergent validity, shared dimensionality, and linkages to the five-factor model. *Front Psychol, 5*, 1164. doi:10.3389/fpsyg.2014.01164

Sperduti, M., Martinelli, P., & Piolino, P. (2012). A neurocognitive model of meditation based on activation likelihood estimation (ALE) meta-analysis. *Consciousness and Cognition, 21*(1), 269-276. doi:10.1016/j.concog.2011.09.019

Tomasino, B., Fregona, S., Skrap, M., & Fabbro, F. (2012). Meditation-related activations are modulated by the practices needed to obtain it and by the expertise: an ALE meta-analysis study. *Front Hum Neurosci, 6*, 346. doi:10.3389/fnhum.2012.00346

Vago, D. R., & David, S. A. (2012). Self-awareness, self-regulation, and self-transcendence (S-ART): A framework for understanding the neurobiological mechanisms of mindfulness. *Front Hum Neurosci, 6*, 1-30. doi:10.3389/fnhum.2012.00296

van der Velden, A. M., Kuyken, W., Wattar, U., Crane, C., Pallesen, K. J., Dahlgaard, J., . . . Piet, J. (2015). A systematic review of mechanisms of change in mindfulness-based cognitive therapy in the treatment of recurrent major depressive disorder. *Clin Psychol Rev, 37*, 26-39. doi:10.1016/j.cpr.2015.02.001

Visted, E., Vollestad, J., Nielsen, M. B., & Nielsen, G. H. (2015). The Impact of Group-Based Mindfulness Training on Self-Reported Mindfulness: a Systematic Review and Meta-analysis. *Mindfulness, 6*(3), 501-522. doi:10.1007/s12671-014-0283-5

Wallace, B. C., Small, K., Brodley, C. E., Lau, J., & Trikalinos, T. A. (2012). *Deploying an interactive machine learning system in an evidence-based practice center: abstrackr.* Paper presented at the Proceedings of the 2nd ACM SIGHIT International Health Informatics Symposium.

Whitfield, T., Barnhofer, T., Acabchuk, R., Cohen, A., Lee, M., Schlosser, M., . . . Marchant, N. L. (2021). The Effect of Mindfulness-based Programs on Cognitive Function in Adults: A Systematic Review and Meta-analysis. *Neuropsychol Rev*. doi:10.1007/s11065-021-09519-y
